# Exploring vaginal microbiome: from traditional methods to metagenomic next-generation sequencing—a systematic review

**DOI:** 10.3389/fmicb.2025.1578681

**Published:** 2025-08-14

**Authors:** Siying Liu, Yuxin Chen, Kunxiu Zhang, Dan Tang, Ji Zhang, Yuxin Wang, Jiaqi Zhao, Diyan Li, Tao Wang

**Affiliations:** ^1^School of Pharmacy, Chengdu University, Chengdu, China; ^2^School of Computer Science, Chengdu University, Chengdu, China; ^3^School of Basic Medical Sciences, Chengdu University, Chengdu, China

**Keywords:** vaginal microbiome, vaginal health, mNGS, personalized treatment, clinical applications

## Abstract

Recent research has highlighted the vaginal microbiome as a crucial factor in women's health and fertility. The growing recognition of its significance has intensified the focus on studying the female reproductive tract's microbial ecosystem. While various analytical methods exist for examining the vaginal microbiome, metagenomic next-generation sequencing (mNGS) has emerged as an auspicious approach. This study examines how mNGS technology can be applied to analyze vaginal microbiota. We begin by exploring the relationship between vaginal bacterial communities and women's health, followed by a comparative analysis of metagenomics against other detection methods, highlighting their respective strengths and limitations. The paper systematically reviews different detection techniques, examining their fundamental principles, constraints, and advantages. Several factors can affect data quality, including sampling procedures, contamination issues, and PCR amplification errors. We suggest implementing third-generation sequencing (TGS) to address these challenges to enhance reproducibility and read length, utilizing single-molecule sequencing (SMS) to eliminate PCR amplification-related errors, and integrating multiple analytical approaches to provide comprehensive insights. In summary, mNGS technology allows us to collect valuable information at a lower cost, and it remains a leading method for detecting female reproductive tract microbes. The goal of this review is to describe the principle, benefits and drawbacks, and application areas of mNGS, as well as to serve as a reference for research into female reproductive tract microbial detection methods, promote the improvement of mNGS in the detection of female reproductive tract microbial technology, and ensure the health of the female reproductive tract.

## 1 Introduction

### 1.1 Importance of studying the vaginal microbiome

The human body is a holistic organism made up of various microorganisms, including the vagina, which is a vast microecosystem containing billions of microorganisms. The microorganisms in the vagina play an essential role in protecting the female reproductive tract health and reducing gynecological infections (Nori and Vaginal, 2023). The disruption of the vaginal ecosystem contributes to the overgrowth of pathogens, which causes complicated vaginal infections, predisposing factors such as menses, pregnancy, sexual practice, uncontrolled usage of antibiotics, and vaginal douching can alter the microbial community (Chee et al., 2020).

In healthy people, the vaginal microbial environment has low diversity of bacteria compared to the intestinal microflora (Ravel et al., 2011; Collins et al., 2017). Numerous studies suggest that a *Lactobacillus*-dominated community is probable to be discovered in the healthy-state vaginal, *Lactobacillus* abundance acidifies the vaginal medium to the average pH, 3.5 ± 0.2 (O'Hanlon et al., 2013), and higher vaginal pH (less acidic) has been observed in diseased-state vagina (Ravel et al., 2011; Ceccarani et al., 2019; Brooks et al., 2017a; Zheng et al., 2019; Drell et al., 2013), the research study of 396 North American asymptomatic women from four ethnic groups revealed that single or multiple *Lactobacillus* species dominated a majority of vaginal microbiomes. *Lactobacilli* have been confirmed to constitute the first line of defense against pathogens, the moderate abundance of L. curl and L. inux (~80%) (I-B: 54.35% and III-B: 57.73%) were found to be beneficial for pregnancy outcome, *Lactobacillus* showed higher abundance in women with premature ovarian insufficiency, which can reduce the number of pathogens in the vagina and thus is related to vaginal health (Kalia et al., 2020; Wang T. et al., 2024). They are divided into five CSTs. CSTs I, II, III, and V are dominated by *L. crispatus, L. gasseri, L. iners*, and *L. jensenii*. In contrast, CST IV refers to the high diversity of the microbial community characterized by obligate anaerobic bacteria (Chen X. et al., 2021). The vaginal microbiome (VMB) in normal reproductive-aged women also constitutes the fungal part designated as the “vaginal mycobiota” (Drell et al., 2013). The predominant part of this mycobiota was occupied by *C. albicans* (72%−91%), *Candida albicans*, an opportunistic fungal pathogen, grows in 20% of women without causing any overt symptoms. Yet, it is one of the main causes of infectious vaginitis (Bradford and Ravel, 2016), which is followed by non-albicans Candida (NAC) species, including *C. glabrata, C. tropicalis*, and *C. para psilosis* (Barousse et al., 2004).

**Figure 1:**
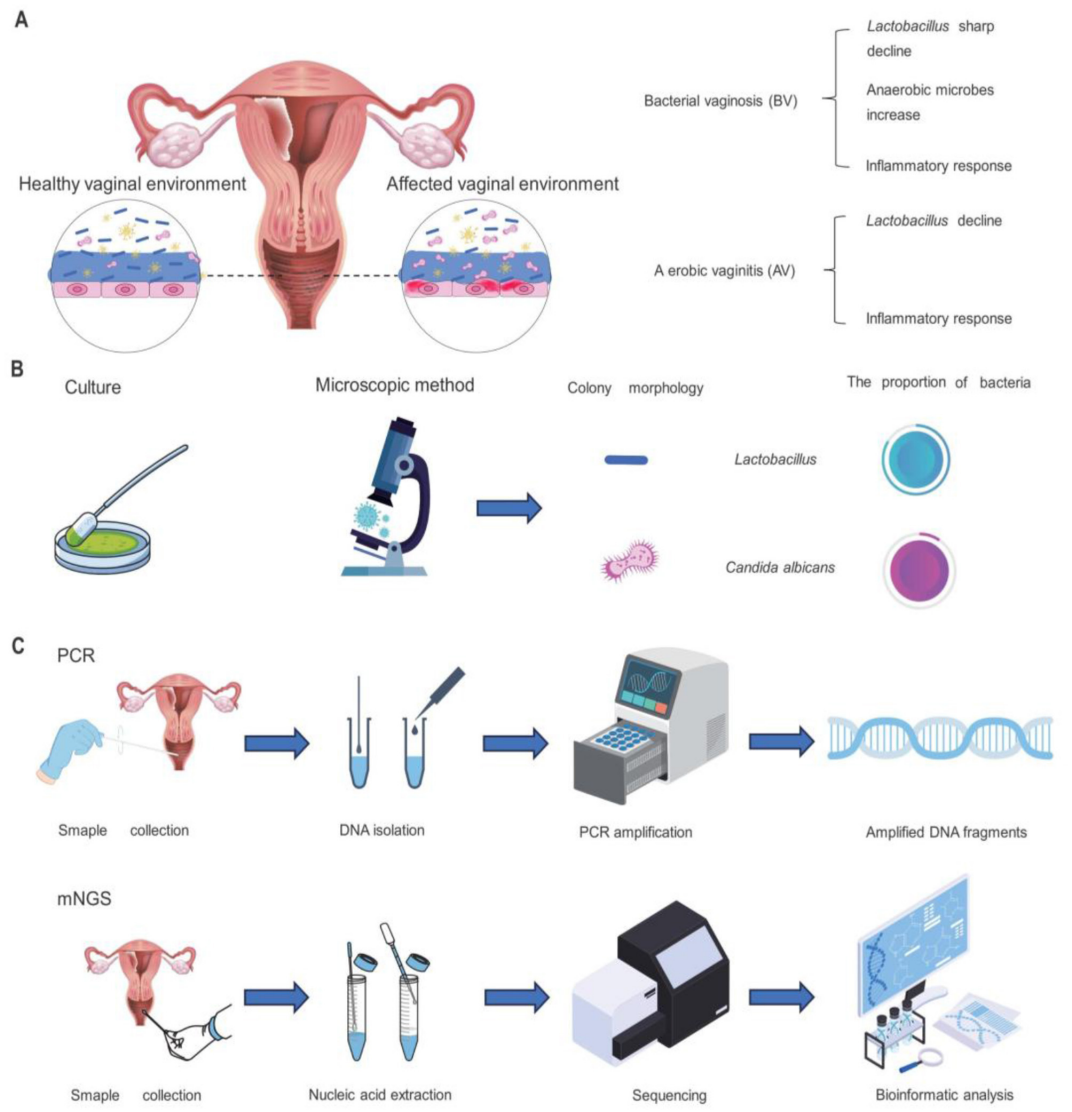
Vaginal symptoms of AV or BV infection in women and methods of vaginal microbiome detection. **(A)** Changes in the female reproductive tract environment following AV or BV infection. **(B)** Procedures and outcomes of culture and microscopic investigation for the detection and analysis of female vaginal microorganisms. **(C)** Steps of PCR detection and mNGS for microbiome detection.

Vaginal microecological disorders will cause changes in the cleanliness and pH of the vagina, which will quickly result in vaginitis and build an ideal environment for the ascending infection of endogenous and exogenous pathogens, which leads to the occurrence of pelvic inflammatory disease and further leading to intrauterine adhesions (IUA) (Paavonen and Brunham, 2019). Bacterial vaginosis (BV) is caused by a loss or sharp drop in the total number of *Lactobacillus* and a corresponding substantial increase in the concentration of anaerobic bacteria in women, and it is a relatively widespread vaginal microbiota problem among women of reproductive age worldwide (Figure 1A). BV sufferers were shown to rely on nitrogen sources rather than carbon sources for their energy needs. Amino acid catabolism produces amines that cause fishy odors; hence, the patient's secretions frequently smell like fish (Kalia et al., 2020).

In addition to that, vulvovaginal candidiasis (VVC) and aerobic vaginitis (AV) are recognized as risk factors for infertility (Figure 1A). Vulvovaginal candidiasis is estimated to be the second most common cause of vaginitis after bacterial vaginosis (Lopez, 2013), exceptionally common 3 in 4 women will be affected at least once over their lifetime (Hurley and De Louvois, 1979), *Candida albicans* accounts for 85% to 90% of cases, asymptomatic carriage rates for *C. albicans* in healthy women are estimated at around 20% (Goldacre et al., 2010; Drake and Maibach, 1973). Recurrent vulvovaginal candidiasis affects nearly 138 million women globally each year. The prevalence of aerobic vaginitis is 7% to 12%, especially during pregnancy, and is associated with adverse pregnancy outcomes. Patients with AV had a reduced or absent number of *Lactobacilli* in the vagina, increased secretion, and a more severe inflammatory response. AV is similar to BV, but the inflammatory response is more intense, involving increased cytokines such as IL-1β, IL-6, and IL-8 (Peebles et al., 2019). A recent study analyzed the vaginal microbiota of 1,411 women and found an association between vaginal microbial composition and fertility. This finding suggests that detecting vaginal microbiome composition may help diagnose infertility and potentially improve the success rate of *in vitro* fertilization. Vaginal microecological detection can be divided into two parts: functional detection and morphological detection (Mahajan et al., 2022), these two methods are complementary to each other and can comprehensively evaluate the vaginal microecological status (Chen H. et al., 2021; Sharma et al., 2021; Hong et al., 2016). Functional test: functional testing assesses the functional status of vaginal microbes by detecting their metabolites and enzymatic activities. For example, Vaginal PH value, this index can reflect the PH of the vaginal microecological environment, and then determine whether there is a microecological imbalance (O'Hanlon et al., 2019; van den Tweel et al., 2022; Heinze et al., 1989; Zodzika et al., 2011), Examination of hydrogen peroxide concentration: it is an index of ecological dominant bacteria (Tomás et al., 2003; Pashaian and Oganesian, 2011). Leucocyte esterase test: it is an indicator of host reaction (Arango-Sabogal et al., 2019; Chacko et al., 1996; Mårdh et al., 2003; Abbasi et al., 1985), Examination of sialidase activity: the index of pathogenic bacteria is reflected. Morphological detection: vaginal secretions wet microscopy: this detection method is mainly to observe whether there are clues in vaginal secretions, trichomonas vaginalis or white blood cells, to diagnose inflammation (Abbasi et al., 1985; Roy et al., 2023; Patil et al., 2012). Gram stain smear microscopy: this detection method mainly evaluates the dominant bacterial community, performs leucorrhea score, and observes whether there is false mycelium or spores of *candida* (Danby et al., 2021; Schmidt and Hansen, 2001). In clinical work, the two detection methods are usually combined, with morphological detection as the primary and functional detection as the auxiliary so doctors can make a comprehensive judgment. This approach could improve diagnostic accuracy, and help doctor's better plan treatment.

Recent studies have demonstrated a significant correlation between the composition of the vaginal microbiota and the outcomes of *in vitro* fertilization (IVF) treatments. Notably, a vaginal microbiota dominated by *Lactobacillus crispatus* is associated with higher implantation and pregnancy rates. A study utilizing 16S rRNA sequencing classified cervical microbiota into three types: CMT1 (dominated by L. crispatus), CMT2 (dominated by *L. iners*), and CMT3 (dominated by other bacteria). The results indicated that the biochemical and clinical pregnancy rates were significantly higher in the CMT1 group compared to CMT2 and CMT3, suggesting that a *L. crispatus*-dominant microbiota may serve as a predictive marker for successful IVF outcomes (Guan et al., 2023). Conversely, a vaginal microbiota dominated by anaerobic bacteria such as *Gardnerella vaginalis, Atopobium vaginae*, and *Prevotella* species has been associated with adverse IVF outcomes. Systematic reviews and meta-analyses have found that vaginal dysbiosis, including bacterial vaginosis and aerobic vaginitis, correlates with reduced clinical pregnancy rates and increased risks of early pregnancy loss in IVF patients (Maksimovic Celicanin et al., 2024). Furthermore, studies have indicated that both excessively high and low abundances of Lactobacillus may negatively impact pregnancy outcomes. A study involving 1,411 women found that a moderate abundance (~80%) of *L. crispatus* and *L. iner*s was associated with higher pregnancy rates, whereas abundances exceeding 90% or falling below optimal levels could potentially reduce the likelihood of successful pregnancy. In summary, maintaining a balanced vaginal microbiota, particularly one dominated by *L. crispatus*, may enhance IVF success rates. Therefore, assessing the vaginal microbiota composition prior to IVF treatment and implementing interventions, such as probiotic supplementation, when necessary, could serve as effective strategies to optimize reproductive outcomes.

### 1.2 Brief overview of methods for analyzing vaginal microbiomes

The morphological examination is usually used for vaginal microbiology testing, performed by a smear of vaginal secretions, vaginal Gram staining (Nugent score and Ison-Hay criteria), culture-based, and pathogenic microorganisms of the vaginal flora, for determining the density, diversity, and dominant species (Figure 1B). The classical analysis employs methods contingent upon cultural practices; indeed, after a defined period of cultivation, various bacterial species can be distinguished through characteristic cell staining, morphological examination, or observed biochemical activities (Hok and Loen, 1967). Although these methods can provide some information, they have certain limitations, such as the inability to detect microorganisms that are difficult to culture. It is also important to note that the Nugent score method does not differentiate particular *Lactobacillus* species (Muzny et al., 2023).

Direct probe assays add the DNA probes into the vaginal fluid specimen; the probes then attach to specific sequences of the particular bacterium, allowing the detection of many germs in a single specimen (Coleman and Gaydos, 2018). NAATs, like PCR, can detect as few as one bacteria in a vaginal specimen (Coleman and Gaydos, 2018). Quantification of bacterial species in the vaginal microbiota by qPCR is a popular tool for identifying and measuring specific vaginal microorganisms (Jespers et al., 2012; Fredricks et al., 2009; Zozaya-Hinchliffe et al., 2010). The 16S rRNA gene was selected to assay bacterial communities (Woese and Fox, 1977). Scientists developed universal primers that amplify a particular gene segment, making it possible to use PCR to analyze the total DNA collected from an entire bacterial population in a community sample (Baker et al., 2003). In practice, these primers achieve over 95% coverage, missing only a small proportion of microorganisms in the community depending upon the primer selected (Sofie et al., 2017).

Metagenomic next-generation sequencing (mNGS) is increasingly used in clinical laboratories for indiscriminate microbial culture and independent diagnosis. This technology can identify rare, novel, hard-to-detect, and co-infected pathogens directly from clinical samples (Figure 1C). mNGS is characterized by its high sensitivity; it can effectively improve the sensitivity of hard-to-detect microorganisms and pulmonary co-infections. It also shows excellent potential for predicting antibiotic resistance, providing new diagnostic evidence to guide treatment options, and providing important information for clinical treatment (Gu et al., 2019). Recent publications have addressed the subject of metagenomic analysis pipelines and the challenges encountered, yet there is a paucity of in-depth exploration of host DNA contamination and species misclassification. It is evident that tools such as Kraken2 and MetaPhlAn are of pivotal significance. Kraken2 employs k-mer-based classification with databases such as RefSeq, utilizing confidence score (CS) adjustments (e.g., 0.2–0.4) to balance classification rate and precision. However, higher CS (≥ 0.6) can significantly reduce classification accuracy for smaller databases like Minikraken. MetaPhlAn4 integrates reference genomes and metagenome-assembled genomes (MAGs) via species-level genome bins (SGBs), leveraging unique marker genes to improve taxonomic profiling, especially for uncultured species, and outperforms alternatives in synthetic evaluations.

The challenges posed by these issues include the misclassification of species due to taxonomic ambiguity or incomplete databases. MetaPhlAn4 mitigates this via SGB clustering, while Krake2′s CS controls k-mer agreement to reduce false positives. Host DNA contamination, although less frequently discussed, necessitates preprocessing steps such as host read filtering, which are implicit in workflows but not explicitly detailed. In clinical settings, interpretation thresholds vary: Kraken2 employs CS = 1.0 for pathogen detection with the objective of minimizing false alarms (Liu et al., 2024), while MetaPhlA4′s marker-based approach facilitates strain-level profiling for the discovery of biomarkers (Blanco-Míguez et al., 2023). The management of ambiguity necessitates the curation of databases (for instance, GTDB r202 to ensure taxonomic consistency) and multi-tool validation. However, there is a paucity of standardized protocols for clinical applications. The challenge of balancing computational feasibility (for example, the size of RefSeq vs. the efficiency of Minikraken) and accuracy remains significant. The performance of larger databases such as nt is enhanced, but greater demands for resources are placed upon them.

## 2 Methodological comparison

### 2.1 Culture

#### 2.1.1 Mechanism and application of the culture method

The conventional method for examining vaginal microbiota relies on two primary techniques: wet mount microscopy and discharge culture. During wet mount examination, clinicians place a sample of vaginal discharge on a glass slide and examine it microscopically to evaluate bacterial and fungal characteristics, including their morphology, quantity, and types of microorganisms present. This can help doctors get an initial understanding of the state of the vaginal microbiome (Savicheva, 2023). The culture process involves inoculating vaginal discharge specimens into specialized growth media. This cultivation method enables the identification and quantification of various bacterial and fungal species, providing detailed insights into the vaginal microbiota composition (Villaseca et al., 2015). The longitudinal study with continuous sample collection tracked the changes for 6 months. All samples were obtained during the follicular phase of the menstrual cycle (days 7–14) to minimize hormonal changes as much as possible.

Although culture-based diagnostic methods are slower than modern molecular techniques, they remain the preferred approach for detecting certain fungal pathogens due to their ability to perform antimicrobial susceptibility testing (Willinger, 2017). Culture-based methods have the advantages of a more precise quantitative analysis and the ability to isolate the targeted foodborne bacteria, but they also have the disadvantage of providing information that is mainly focused on the target microorganisms (Wang et al., 2017). Vaginitis/vaginal infections are among the most prevalent gynecological conditions encountered in clinical practice. The primary culprits include yeast infections (most commonly caused by *Candida albicans*, the protozoan *Trichomonas vaginalis*, or an imbalance in vaginal bacteria leading to bacterial vaginosis. The incidence of each varies significantly across different patient demographics. Since symptoms and physical exams alone are insufficient for a definitive diagnosis, laboratory testing plays a crucial role. For detecting *Trichomonas vaginalis*, culture-based methods remain the gold standard due to their high sensitivity. Meanwhile, bacterial vaginosis can be diagnosed when at least three of the following criteria are met: (1) a thin, homogeneous discharge, (2) vaginal pH exceeding 4.5, (3) a distinct fishy odor upon amine testing, and (4) the presence of clue cells under microscopy (Spiegel, 1989).

#### 2.1.2 Culture-based techniques: strengths and limitations

**Figure 2:**
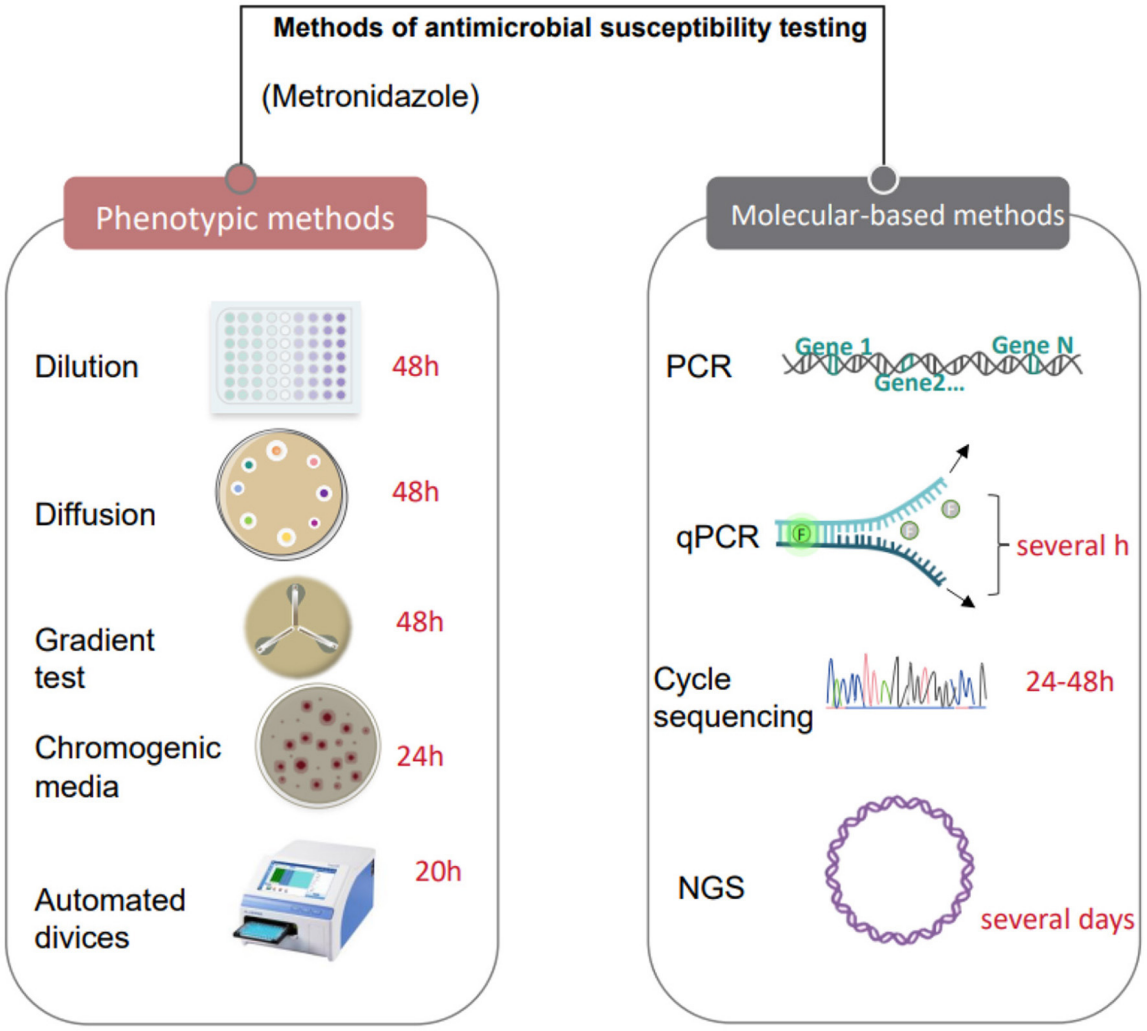
Antimicrobial susceptibility testing methods based on culture and molecular technologies.

Microbial culture technology is a crucial experimental method in microbiology through which various microorganisms can be isolated, cultivated, and studied (Mahler et al., 2021). This technology has many advantages; it can isolate microorganisms from samples through appropriate media and culture conditions, and culture and propagation *in vitro*, and can detect and analyze different physiological and biochemical characteristics of varying microbe (Preksha et al., 2021), such as morphological characteristics, metabolic characteristics, growth rate, product generation ability, etc., to further study the biological characteristics and application value of microorganisms (Marcellino et al., 2015). It can also avoid cell death and atrophy by regularly changing the medium and adjusting the culture conditions to increase the number of microorganisms and get more of the product we want (Wan et al., 2023). In terms of vaginal microbial detection, culture technology can provide more accurate microbial quantification and identification results and can detect some common pathogenic bacteria and microorganisms (Schmidt et al., 2015). In addition, the culture technique can also be used to conduct antimicrobial susceptibility experiments to test the sensitivity and resistance of different antibiotics to vaginal microorganisms, guiding clinical treatment (Sood et al., 2022; Cheng et al., 2005) (Figure 2). Compared with other detection methods, the culture technique has high specificity and sensitivity, which can effectively detect the presence and number of microorganisms and provide an essential basis for the evaluation and treatment of vaginal microecology.

However, microbial culture technology has limitations, such as the inability to detect difficult cultivated microorganisms (Ignyś et al., 2014), which require special cultivation conditions, such as anaerobic conditions or specific nutrients, which make these microorganisms challenging to grow in the laboratory, limiting further research and application of these microorganisms (Erokhin et al., 2022). Microorganisms may behave differently under cultural conditions than in their natural environment, affecting the reliability of the study's conclusions (Li et al., 2023). Traditional microbial culture techniques usually take a long time to obtain results, which is unsuitable for microbial detection and identification in urgent situations. Still, the new culture technology requires professional skills, is more complex to operate, and requires professional training. It is also challenging to ensure the purity of microbial cultures during the cultivation process, and they may be contaminated by other microorganisms (Margolles and Ruiz, 2021). Finally, using some special culture conditions and culture media will increase the cost of culture and limit large-scale application.

In summary, among the currently implemented culture methods, simulating natural environmental conditions and maintaining the interrelationship between microbial populations is the key to improving the cultivability of microorganisms in the environment (Peng et al., 2021). Therefore, the research on microbial culture technology should mainly focus on this aspect for in-depth improvement and development. In addition, we need to select the appropriate technology according to the specific research purpose, time requirements, economic costs, experimental conditions, and other factors.

### 2.2 Polymerase chain reaction (PCR)

Conventional detection methods have limited ability to identify microbial species. With the advancement of technology, Polymerase chain reaction (PCR) technology has been introduced into the detection of vaginal microbiota. These changes have had a profound impact on the study of vaginal microbes (Mortaki et al., 2020).

PCR is a molecular biology technique in which specific DNA fragments are rapidly amplified *in vitro* through specific primers and DNA polymerases (Demkin et al., 2017). The application of PCR technology in the detection of vaginal microbiota mainly includes the detection of specific pathogens, the quantitative analysis of microbial communities, and the assessment of microbial diversity (Kalra et al., 2007). Through high-throughput PCR, the vaginal microbial community can be quantitatively analyzed, revealing the composition and changes of the microbial community. The basic process of PCR detection is sample preparation and extraction, primer design and synthesis, PCR amplification reaction, and result analysis and interpretation. With the development of technology, various variants of PCR have gradually been derived, such as quantitative real-time PCR (qPCR), multiplex PCR, nested PCR, etc. The qPCR detection process differs from conventional PCR technology in that fluorescent dyes or fluorescently labeled primers are added during PCR amplification (Kubista et al., 2006). The progress and amount of PCR amplification can be monitored in real-time through the strength of the fluorescence signal, and the amplification curve can be drawn (Arya et al., 2005). The analysis curve allows for the quantification of the DNA of interest (Mattei et al., 2019; Artika et al., 2022). QPCR has the characteristics of high sensitivity, high specificity, and high accuracy (Klein, 2002; Harshitha and Arunraj, 2021). Multiplex PCR can detect multiple target DNA sequences simultaneously in the same PCR reaction (Sahu et al., 2019; Khaki et al., 2023), and the detection process is the same as traditional PCR technology. In multiplex PCR, amplified products of different target sequences may have different lengths or fluorescent labels, allowing them to be distinguished within the same electropherogram or fluorescent signal (Nell et al., 2022). Multiplex PCR allows for the simultaneous detection of multiple microorganisms in the same reaction, improving detection efficiency and saving samples and reagents.

PCR technology possesses the advantages of strong specificity, high sensitivity, saving time and raw materials, high efficiency and rapidity, and simple operation. At the same time, there are also the following disadvantages: different probes need to be synthesized according to other sequences, which increases the cost of experiments. Quantitation is susceptible to the performance of reagents and enzymes. The test results make determining the actual amplification characteristics difficult, and there is a high probability of false positives (Zhu et al., 2020).

### 2.3 16S rRNA

The 16S rRNA gene sequence is ~1,550 base pairs in length and is comprised of a variable region and a conserved region. The gene is sufficiently large to allow for the measurement of 16S rRNA gene intragenic variation, which can be used for the purpose of differentiation and statistical analysis. In addition, the 16S rRNA gene can be compared with the 16S rRNA genes of all bacteria, as well as with the 18S rRNA genes of eubacteria and true eukarya. In the 1960s, (Dubnau et al. 1965) it was observed that the 16S rRNA gene sequence of the genus Listeria exhibited a high degree of conservation. Following Woese's pioneering research, this sequence has become a widely utilized tool for the identification and classification of bacteria. The 16S rRNA gene is arguably one of the most conservative genes, despite the ambiguity surrounding its absolute variability. It serves as a significant indicator of the evolutionary distance and relatedness of various biological organisms. The 16S rRNA gene sequence has been utilized in the field of microbiology for the purpose of bacterial identification and the delineation of relationships at the species and strain levels. Its application in clinical microbiology is well-documented. The sequence exerts a significant influence on the relationship between more distant related lineages. The 16S rRNA gene is a universal feature of bacteria, thus enabling the measurement of relationships between all bacterial species (Nygaard et al., 2020; Brinkmann et al., 2021). It is generally accepted that the comparison of 16S rRNA gene sequences enables the differentiation of most bacterial species at the species level. In addition, the classification of strains at multiple levels, including the present species and subspecies levels, is possible. The utility of 16S rRNA gene sequencing is occasionally contingent on the presence of multiple well-known species with similar or identical sequences.

In general, 16S rRNA gene analysis is not considered adequate for the purpose of comparing the prevalence of specific strains or for the identification of strains with specific virulence factors, due to the absence of sufficient variation in the gene region in question (Clarridge, 2004). A comparison of genetic sequences can provide a clear definition of the species or strain of bacterium in question. However, the author's approach of establishing a “species” match for these sequences is not consistent with current standards. In these studies, the definition of “match” for species is not more than 99% similar (< 1% different). In the case of Vibrio cholerae, the genetic sequence can include up to six copies of the 16S rRNA gene, with a difference of up to 1. This indicates that there is heterogeneity within the 16S rRNA gene of the Mycoplasma species, and that the method should not be used for the purpose of species identification (Janda and Abbott, 2007).

### 2.4 Metagenomics next generation sequencing (mNGS)

mNGS technology stands out in the detection of vaginal microbiomes for its broad-spectrum capabilities, high sensitivity, and rapid detection. Compared with traditional methods, mNGS can analyze the structure and composition of vaginal microbial communities more comprehensively. This diagnostic method can directly perform unbiased high-throughput sequencing on the microbial genomes in clinical samples, and can simultaneously detect multiple microorganisms such as bacteria, viruses, fungi, and parasites (Chiu and Miller, 2019), including hard-to-culture and uncommon organisms (Hong et al., 2016). Through precise analysis of the vaginal microbiota, mNGS can diagnose various vaginal diseases more accurately. Meanwhile, its rapid result delivery is especially valuable for diagnosing acute infections. Furthermore, mNGS can predict antibiotic resistance based on microbial genome information, aiding clinical treatment decisions.

mNGS refers to metagenomic next-generation sequencing technology, which does not rely on traditional microbial culture, directly extracts all nucleic acids in the specimen for high-throughput sequencing, and compares the screened data with the pathogen database through the analysis of biological information after removing the human sequence, to obtain the species information of suspected pathogenic microorganism. Due to the development of newer sequencing assays, assessing all microorganisms in a sample through a single mNGS analysis has become feasible (Diao et al., 2021). The mNGS method is a valuable supplement to conventional culture methods, which perform a higher positive rate and sensitivity (Chen H. et al., 2021). It has proven a potentially formidable instrument in various fields, including clinical diagnosis, hospital epidemiology, microbial evolutionary biology, and exploring host-pathogen interactions (Handel et al., 2021). In the field of clinical microbiology, metagenomic sequencing is referred to as clinical metagenomics. Unprecedented ability to capture total nucleic acid of any microorganism in parallel from a single clinical sample. mNGS provided promising support to rapid pathogen diagnosis (Chen M. et al., 2024). Using high resolution can provide a more decadent visual hierarchy, making details more refined and creating a sense of three-dimensional space. High-resolution techniques can extract richer biological information, more significant amounts of data, and more details.

Metagenomic sequencing technology can overcome the shortcomings of unbiased methods, such as the typical PCR assay is difficult to quantify and has various limitations (Lee et al., 2023). Sometimes, unsuitable reaction conditions will affect the effect of the PCR reaction, resulting in inaccurate results and a relatively low detection rate.

### 2.5 Third generation sequencing (TGS)

The revolution in large-scale parallel sequencing began in 2005 with the introduction of Roche's 454 pyrosequencing system, which ushered in next-generation sequencing (NGS) technology (Margulies et al., 2006). This alternative to traditional pyrosequencing enabled DNA sequencing to be performed in a massively parallel manner, marking the birth of the first high-throughput sequencing platform. While NGS transformed the field by delivering unprecedented sequencing power, remarkable depth, and impressive accuracy, it wasn't without its drawbacks—chief among them being the production of short read lengths. The called “short-read sequencing” inherent to all NGS platforms necessitated specialized bioinformatics tools and complex post-processing workflows, complicating the handling of high-throughput data and extending overall analysis times. These limitations were eventually overcome with the advent of third-generation sequencing (TGS), a groundbreaking approach that signaled the dawn of a new era in sequencing. At the same time, we compared the sequencing principle, sequencing read length, advantages, and disadvantages of the three sequencing methods (Table 1).

**Table 1:** Comparison of three generation sequencing technologies.

**Detection method**	**Technical platform**	**Principle of sequencing**	**Read length**	**Advantages**	**Limitations**
The first generation sequencing	Sanger	Chain-terminating sequencing	600–1,000 bp	Long reads; high accuracy; good ability to deal with repetitive and homopolymer regions.	Low throughput; high cost of Sanger sample preparation; making massively parallel sequencing prohibitive.
The second generation sequencing	Roche/454	Pyrosequencing	200–400 bp	Longest read lengths among the second-generation; high throughput.	Challenging sample preparation; hard to deal with repetitive/homopolymer regions.
	Illumina	Sequencing by synthesis	2 × 150 bp	Very high throughput	Short reads; genome assembly is difficult due to complex data processing and short sequence generation
	ABI/Solid	Sequencing by ligation	25–35 bp	High throughput; low cost.	Long sequencing mns (days); short reads, resulting in difficulties in subsequence data analysis and genome assembly.
The third generation sequencing	PacBio SMRT	Sequencing by synthesis/DNA polymerase	~1,000 bp	Long average read length; no amplification of sequencing fragments; longest individual reads approach 100 kb.	Low accuracy; dependence on DNA polymerase activity.
	Nanopore	Electronic signals sequencing/exonuclease	Maximum record 2.2 M	Over-long read; electronic sequencing; portable.	High sequencing error

The TGS method introduces two fundamental and distinctive features: single-molecule sequencing (SMS) and real-time sequencing, which enable nucleotide (DNA or RNA) analysis without PCR amplification of the template. This approach also facilitates immediate data processing as sequencing occurs. A major advancement of TGS lies in its ability to sequence genetic material directly, bypassing the need for template amplification—thereby minimizing the biases typically introduced by PCR during library preparation. In contrast to the relatively brief read length (maximum 600 nt) of NGS platforms, TGS platforms have been shown to exhibit a longer read length, with an average length in excess of 10 kb (van Dijk et al., 2018; Goodwin et al., 2016). The significant increase in the length of generated sequencing reads is considered to be one of the most advantageous features of TGS technology, as it has been demonstrated to markedly enhance the quality of genome assembly and structural analysis of genomes (Michael et al., 2018; Roberts et al., 2013). It can be stated with greater accuracy that the longer the read length, the more representative the sequence of the genome, and according to the analysis of variation, the longer the read length, the more likely it is that the insertion, deletion, and other structural variations in the entire genome will be identified. Consequently, the more continuous the reconstruction of the genetic sequence.

TGS has been demonstrated to facilitate the acquisition of novel insights into the analysis of microbial communities. The TGS platform has been demonstrated to facilitate rapid 16S sequencing procedures, thereby enabling the real-time identification of any target sample's existing bacterial species (Heikema et al., 2020). The employment of full-length 16S rRNA sequencing in the efficient and timely selection of microbial communities facilitates the simplification of analysis and the provision of in-depth strain-level distinguishing information, as well as the current microbial composition's unbiased composition and functional characteristics.

Despite the extensive research and clinical applications of TGS technology, and this technique streamlines the library construction process, it comes with notable drawbacks, including time-consuming sequencing runs, elevated costs, higher error rates, and, not least of all, the production of relatively short reads (averaging ~32 bp) (Athanasopoulou et al., 2021). Due to the inaccuracies observed in the results obtained during the survey period (~15%), it is not recommended to utilize TGS for the precise detection of single-nucleotide polymorphisms (SNP) or point mutations. Nevertheless, the TGS platform's chemical analysis is undergoing enhancement to reduce these error rates and enhance the accuracy of the surveys (Amarasinghe et al., 2020). In consideration of the fact that TGS embodies a pioneering methodology in the domain of surveying, the evolution of downstream analysis tools and algorithms for alternative biological information constitutes a formidable undertaking (Jung et al., 2019).

Comparing TGS with mNGS (Table 2), firstly, the third-generation sequencing (TGS) technology requires a higher current during the sequencing process to maintain the stability of single-molecule sequencing. However, due to its single-molecule sequencing characteristics, current limitation is not as significant as traditional sequencing techniques in the electroporation transfection process of mRNA. Current parameters such as electric field strength and duration have a significant impact on transfection efficiency and cell survival rate. Excessive current may cause cell membrane damage and cell death, while insufficient current may lead to insufficient transfection efficiency. The third-generation sequencing (TGS) technologies have high precision and can provide real-time sequencing at the single-molecule level. They can directly detect RNA modifications and full-length transcripts. The throughput of TGS is usually low, with fewer reads per run but more runs, while NGS has a higher throughput, generating billions of reads per run, making it suitable for sequencing many samples in one run.

**Table 2:** Compare TGS to mNGS regarding accuracy, throughput, read length, cost, and current limitations.

**Parameter**	**TGS**	**mNGS**
Accuracy	High per-read error (5%−15%), but correctable (> 99%)	Ultra-high base accuracy (error rate < 1%), but PCR bias
Throughput	Moderate (50–100 Gb/run)	Extremely high (hundreds of Gb to Tb/run)
Read length	Ultra-long (50–100 kb)	Short (250–300 bp)
Cost	Higher instrument and pre-base cost	Lower cost at high throughput
Current limitations	High error rates, computational intensity	Short-read fragmentation, host contamination

### 2.6 Method comparison: sensitivity, specificity, taxonomic resolution, time to result, cost per sample, can detect novel microbes, and limitations

**Figure 3:**
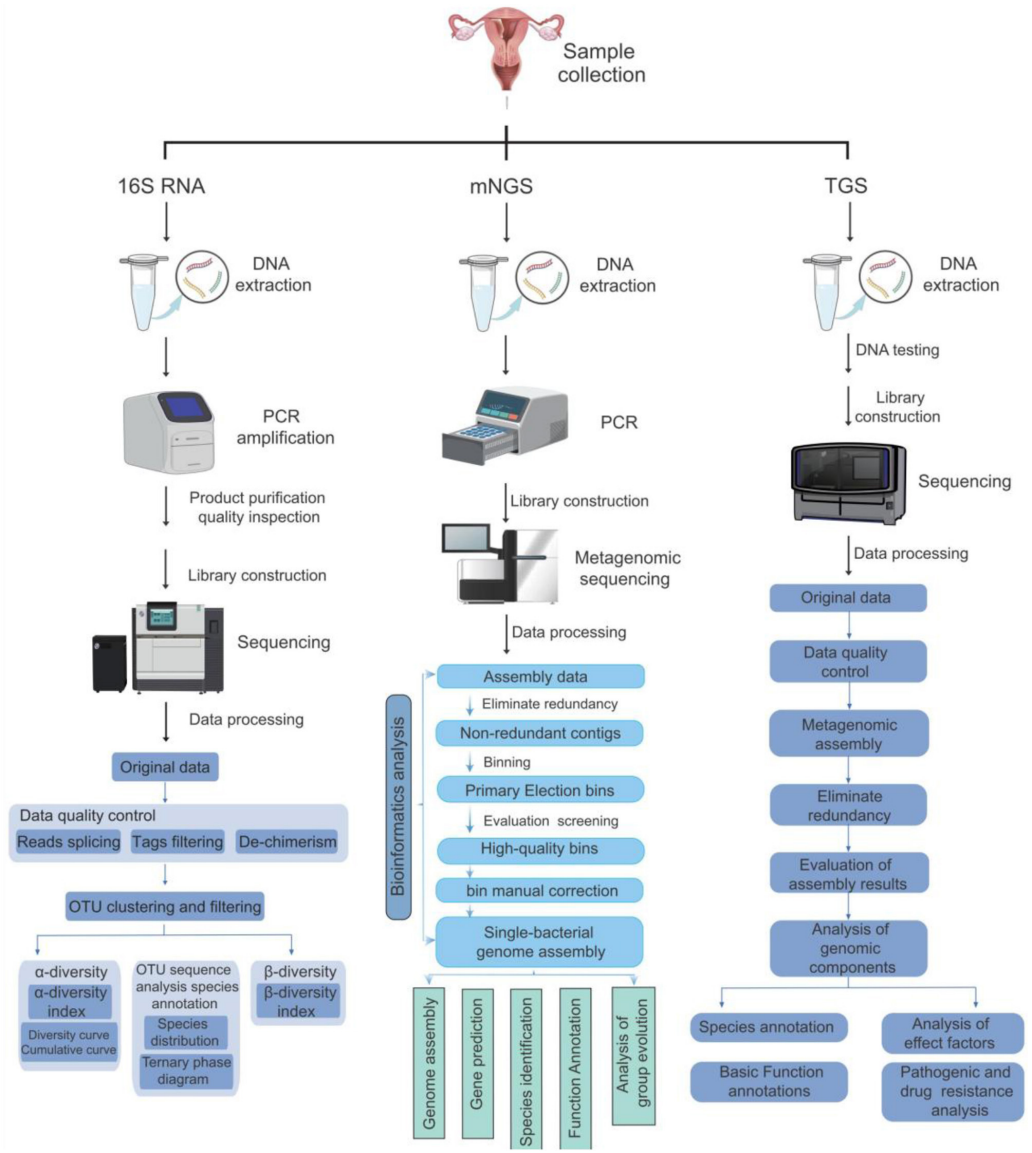
Comparison of three methods for detecting vaginal microorganisms. This figure compares the operation processes, sequencing methods, and bioinformatics analysis processes of the three methods of 16s rRNA, mNGS, and TGS, presenting the differences among the three methods graphically.

Metagenomic next-generation sequencing differs significantly from other traditional methods (e.g., Culture-based techniques and PCR) regarding specificity, sensitivity, breadth of detection organisms, and analysis complexity (Figure 3). mNGS is characterized by unbiased detection (Wang et al., 2019), which can detect all the DNA information in the sample. However, traditional detection methods often target specific microorganisms or genes. Hence, their specificity is high (Liu et al., 2022b), but conventional detection methods make it difficult to detect other unknown microorganisms (Han et al., 2020), which has certain limitations. In terms of sensitivity, mNGS has high sensitivity and can detect a very low abundance of microorganisms in a sample. This is because mNGS does not rely on the process of microbial culture and directly sequences nucleic acids in a sample (Akaçin et al., 2022).

Overall, metagenomic next-generation sequencing offers significant advantages in detecting organisms regarding specificity, sensitivity, and breadth, providing more comprehensive, accurate, and rapid detection results (Punzón-Jiménez and Labarta, 2021; Moreno and Simon, 2019; Gwinn et al., 2019). To this end, an investigation was conducted into the various methods of performing vaginal microbial examinations. The investigation encompassed the following parameters: sensitivity, specificity, diagnostic capacity, duration of action, cost, and the ability to detect pathogens. The investigation focused particularly on the limitations of the methods (Table 3).

**Table 3:** The comparison of the various methods for the examination of vaginal microbes.

**Method**	**Target**	**Sensitivity**	**Specificity**	**Taxonomic resolution**	**Time to result**	**Cost per sample**	**Can detect novel microbes**	**Limitations**	**References**
Culture	Culturable bacteria/fungi	Low	High	Species level	2–7 days	Low	No (only known cultivable bacteria)	Unable to detect anaerobic bacteria/difficult-to-cultivate bacteria; a high rate of missed detection	Abou Chacra et al., 2023; Gauthier et al., 2023
qPCR	Pathogen-specific nucleic acid	High	High	Species/strain level	2–4 h	Low	No (Sequence required to be known)	Only detects preset targets; limited multiple detection capability	Vartoukian et al., 2010; Goodwin et al., 2016
16S rRNA	Universal primers for bacteria	Medium	Medium	Genus level	1–3 days	Medium	Yes (but only in 16s database)	It is impossible to distinguish between live and dead bacteria. PCR amplification deviation	Gauthier et al., 2023; Cummings et al., 2016; Akaçin et al., 2022
mNGS	All microbial nucleic acids	Very high	Low (the host needs to be removed)	Species/strain level	24–48 h	Very high	Yes	The cost is extremely high; Data analysis is complex. The risk of false positives is high	Gu et al., 2019; Chiu and Miller, 2019; Cummings et al., 2016; Akaçin et al., 2022; Diao et al., 2023a
TGS	Real-time DNA/RNA (no amplification)	Extremely high	Medium	Strain level	2–8 h	Very high	Yes	The original read order has a relatively high error rate; it needs bioinformatics correction	Amarasinghe et al., 2020; Scarano et al., 2024

## 3 mNGS: applications, technical, and clinical challenges

### 3.1 Applications of mNGS in vaginal microbiome research

Metagenomic Next Generation sequencing technology (mNGS) is an advanced molecular diagnostic method that can obtain sequence information of microbial nucleic acid fragments in a single run and detect all microbial species and sequences through analysis and comparison (Lecuit and Eloit, 2014). In addition, mNGS can be used to identify and type all pathogens because mNGS does not rely on culture and can retrieve all DNA without bias (Lefterova et al., 2015). Therefore, this technique has significant application value in vaginal microbiome research. The vaginal microbiome is a complex and dynamic microecosystem that changes throughout a woman's life and is dominated by lactobacillus-producing bacteria of the genus *Lactobacillus* (Ma et al., 2012). The mNGS technique enhances our understanding of this ecosystem by providing a high-resolution analysis of the vaginal microbiome. It allows researchers to identify and characterize the microbial diversity within the vagina accurately and how these microbes interact with the host. The application of this technique reveals changes in the vaginal microbiome in different health conditions. By revealing the complexity and dynamic changes of the microbiome, mNGS provides an essential scientific basis for improving women's health.

The primary focus of the metagenomic sequencing data analysis encompassed gene prediction and abundance analysis, specimen annotation, dimensionality analysis of species abundance, LEfSe analysis of differential species between groups, cluster analysis of species abundance, and Metastats analysis of different species between groups (Avershina et al., 2013; Oh et al., 2014; Noval Rivas et al., 2013). The main applications of mNGS in vaginal microbiome research include: microbial diversity Analysis; mNGS can provide a comprehensive assessment of microbial diversity in the vagina, helping researchers (Kero et al., 2023). Disease diagnosis and biomarker discovery: by comparing the vaginal microbiome of healthy women with those with specific gynecological conditions, mNGS helps identify the types of microbes (Aronson and Ferner, 2017). Resistance gene detection: mNGS can detect resistance genes, help to understand the resistance of pathogenic microorganisms, and guide the rational use of antibiotics in clinics (Liu et al., 2022a). Precision Medicine: mNGS helps enable precision medicine targeting the vaginal microbiome by analyzing an individual's microbiome characteristics to provide a personalized treatment plan for patients (Scher et al., 2013).

Metagenomics is defined as the sum of all microbial genomes in the environment and is the sequencing of the sum of all microbial genomes. We focused our research mainly on women of childbearing age (18–45 years old) diagnosed with bacterial vaginosis (BV) through the Nugent score or Amsel criteria, excluding participants who had recently used antibiotics or had concurrent fungal infections. mNGS can be used to detect and analyze the vaginal microbiome by following the steps below (Gu et al. 2019).

Step 1: Sample collection. A microbial sample needs to be taken from the vagina. This usually uses sterile cotton swabs or specialized sampling tools to collect specimens directly from the posterior vaginal fornix, external cervix, and inner cervical area (Subramaniam et al., 2016), and requires strict hygiene and aseptic practices to be followed to avoid contamination by external microorganisms. Step 2: DNA extraction. The collected sample undergoes a series of processes to extract the DNA. This step can be accomplished using a DNA extraction kit or a lab-made extraction method, which includes using chemical reagents to lyse cells, centrifugation, and purification of DNA. The quality of the extracted DNA is critical for subsequent sequencing and analysis (Xiao et al., 2022). Step 3: Metagenomic sequencing. High-throughput sequencing of the extracted DNA, such as second or third-generation sequencing technology, generates a large amount of DNA sequence data representing the genome sequence of all the microorganisms in the sample. Step 4: Bioinformatics analysis. Bioinformatics analysis of sequencing data was performed to understand the diversity, abundance, functional activity, and more of vaginal microbes. This includes steps such as sequence assembly, gene annotation, species classification, functional prediction, and more. With these analyses, the main microbial species in the vaginal microbiome can be identified. Step5: Interpretation and application of results. Based on the results of the analysis, it is possible to interpret the characteristics of the vaginal microbiome, such as microbiota structure, functional activity, etc. This information can help us understand the state of the vaginal microbiome and assess the health of the vagina (Gauthier et al., 2023).

Metagenomic technology has many advantages in the detection and analysis of the vaginal microbiome, providing new methods and means for vaginal microbial detection (Gwinn et al., 2017), which helps understand the diversity and complexity of the vaginal microbiome more comprehensively. It is important to note that when performing vaginal microbial metagenomic sequencing, it is crucial to avoid the influence of contamination and interfering factors.

#### 3.1.1 mNGS-guided personalized therapy based on vaginal microbiome

The mNGS technology provides the possibility for a deeper understanding of the composition and dynamic changes of vaginal microbiota (Tsang et al., 2024). By conducting metagenomic sequencing on vaginal samples, researchers can obtain comprehensive information about the vaginal microbiota, including the presence (Indelli et al., 2021), abundance, and interactions of different bacterial species (Lazarevic et al., 2022). These pieces of information help us better understand the role of vaginal microbiota in women's health and its relationship with gynecological inflammation and other diseases.

The analysis of vaginal microbiota based on mNGS technology can provide an important basis for the development of personalized treatment strategies (Wang et al., 2023). Specifically, doctors can develop targeted treatment plans according to the patient's vaginal microbiome characteristics. For example, for gynecological inflammation caused by imbalanced vaginal microbiota, doctors can restore the balance of the microbiota by using probiotics, antibiotics, or other drugs (Chee et al., 2020). In addition, personalized treatment strategies can also consider factors such as the patient's age, lifestyle, and genetic background to achieve more precise treatment (Wallace and Moodie, 2014).

Furthermore, mNGS technology can also monitor treatment outcomes and the risk of disease recurrence (Huang et al., 2023; Zhang J. et al., 2022). By conducting metagenomic sequencing of vaginal samples after treatment, doctors can understand the recovery of microbial communities and whether there is a potential risk of recurrence (Lian et al., 2024). This helps to adjust the treatment plan promptly and improve the treatment effect and patients' quality of life.

**Figure 4:**
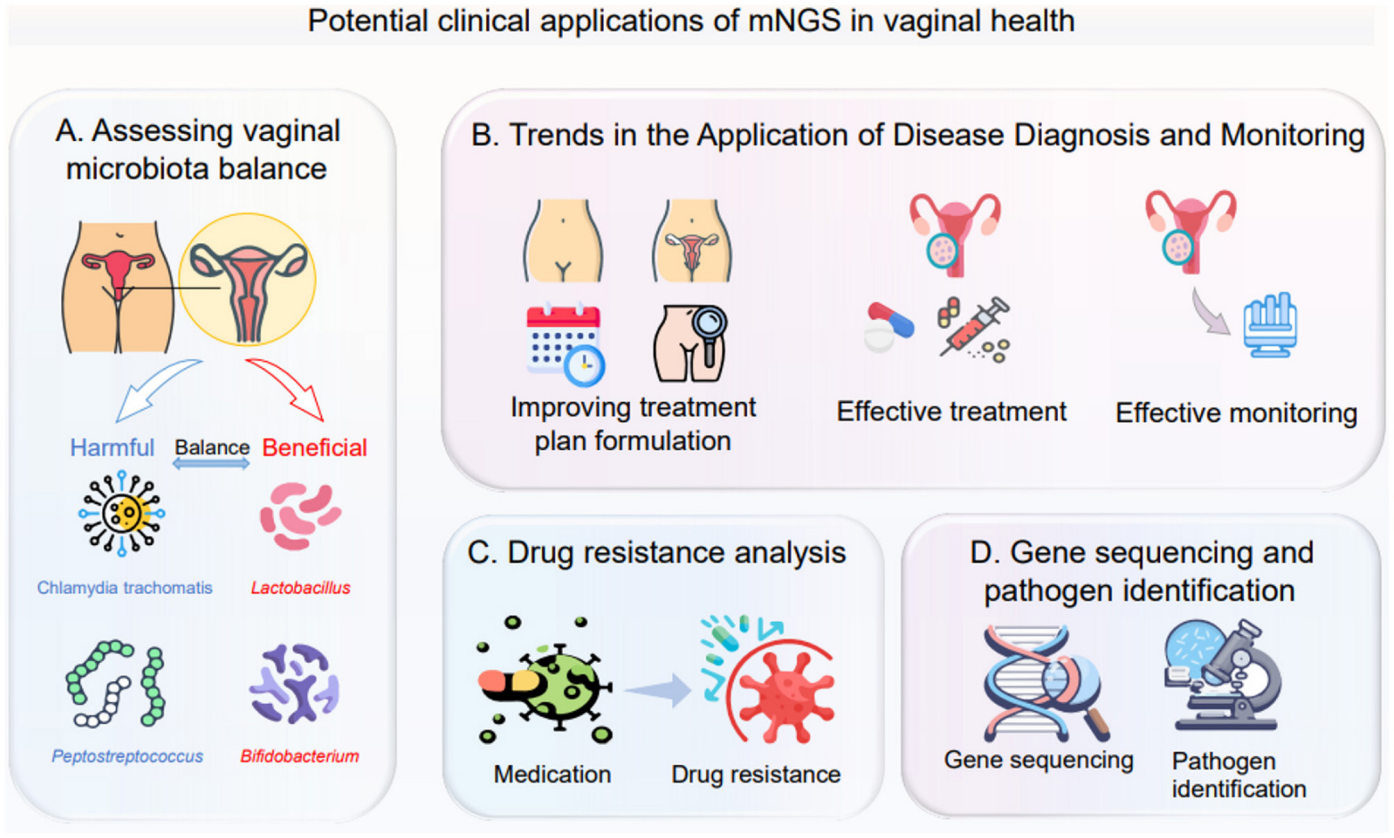
This figure illustrates the potential clinical applications of metagenomic next-generation sequencing (mNGS) in vaginal health, covering the following four aspects: **(A)** Assessing vaginal microbiota balance: analyze the distribution of harmful microorganisms (such as *Chlamydia trachomatis, Peptostreptococcus*) and beneficial microorganisms (such as *Lactobacillus, Bifidobacterium*) to determine the balance status of the vaginal microecology; **(B)** Trends in the application of disease diagnosis and monitoring: from optimizing the formulation of treatment plans, to conducting effective treatment, and then to implementing effective monitoring, it runs through the entire process of disease diagnosis and treatment; **(C)** Drug resistance analysis: observe the changes of microorganisms before and after medication, clarify their drug resistance status, and provide a basis for precise medication; **(D)** Gene sequencing and pathogen identification: use gene sequencing technology to identify pathogens that cause vaginal health problems.

In summary, mNGS technology provides essential support for personalized treatment strategies based on vaginal microbiome (Zhao et al., 2021). By gaining a deeper understanding of the composition and dynamic changes of vaginal microbiota, doctors can develop more precise and personalized treatment plans to meet the individualized needs of patients (Adamczak et al., 2024). With the continuous development and improvement of technology, it is believed that mNGS will make more excellent contributions to women's health in the future (Figure 4) (Tsang et al., 2024).

#### 3.1.2 Impact of biological variability on the consistency of vaginal microbiome sampling

Biological variability is a key factor that affects the consistency and comparability of sampling in vaginal microbiome studies. The composition of the microbiota can vary significantly both between individuals and within the same individual over time, influenced by multiple physiological and behavioral factors.

For instance, the menstrual cycle has been shown to significantly alter the diversity and composition of the vaginal microbiota. During menstruation, microbial diversity tends to increase, with a notable decrease in *Lactobacillus* abundance and an increase in genera such as *Gardnerella* and *Streptococcus*, potentially due to elevated bioavailable iron levels in menstrual blood (Song et al., 2020). Fluctuations in hormone levels also impact microbiome stability. Estrogen and progesterone modulate glycogen levels in the vaginal epithelium, which in turn affects the colonization and persistence of *Lactobacillus* species (Oerlemans et al., 2022). Additionally, the use of hormonal contraceptives, such as oral pills or intrauterine devices, may disrupt the microbial equilibrium, often reducing *Lactobacillus* dominance (Brooks et al., 2017b). Lifestyle factors, including dietary habits (e.g., vegetarianism) and physical activity, are also associated with variability in vaginal microbiota composition and stability (Song et al., 2020).

To improve sampling consistency and data comparability, several strategies should be adopted in study design: (1) Standardizing sampling time—it is recommended to collect samples at a specific stage of the menstrual cycle, such as 48 h after menstruation, to minimize hormonal fluctuation effects (Santiago et al., 2011); (2) Controlling behavioral variables—participants should avoid vaginal intercourse, intravaginal products, or other behaviors that may affect the microbiota at least 48 h prior to sampling (Santiago et al., 2011); (3) Collecting detailed metadata, including information on menstrual phase, contraceptive use, diet, and physical activity, which can help in adjusting for confounding variables during data analysis; (4) Selecting appropriate sampling methods—studies have confirmed that self-collected and clinician-collected vaginal swabs yield comparable microbiome profiles, though the use of lubricants during sampling should be avoided to prevent interference (Forney et al., 2010).

#### 3.1.3 Ethical and data privacy considerations

The integration of metagenomic next-generation sequencing (mNGS) into gynecological diagnostics introduces significant ethical and data privacy concerns. The comprehensive microbial data generated can inadvertently reveal sensitive information about a patient's health status, genetic predispositions, or familial relationships, potentially leading to unintended disclosures. Therefore, obtaining informed consent becomes paramount. Patients must be thoroughly educated about the scope of mNGS, including the types of data collected, potential incidental findings, and the implications of such information. This ensures that consent is both informed and voluntary, aligning with ethical standards in medical practice. Furthermore, safeguarding patient privacy is critical. Implementing robust data protection measures, such as encryption, access controls, and anonymization, is essential to prevent unauthorized access and misuse of sensitive information. These practices are in line with established guidelines that emphasize the importance of confidentiality and data security in medical research and diagnostics. In summary, while mNGS offers advanced capabilities in gynecological diagnostics, it necessitates a careful balance between technological advancement and ethical responsibility. By adhering to stringent informed consent processes and implementing comprehensive data protection strategies, healthcare providers can mitigate the ethical and privacy risks associated with this powerful diagnostic tool.

### 3.2 The merits and challenges of mNGS both technical and clinical

#### 3.2.1 Case studies or research findings highlight the merits of mNGS

mNGS has been extremely useful in studying the vaginal microbiome (Han et al., 2019). The following case studies demonstrate the unique advantages and applications of mNGS technology in vaginal microbiome research: association of vaginal microbiota with bacterial vaginosis: the study found that the vaginal microbiota of healthy women was dominated by *Lactobacillus* bacteria. In contrast, those with bacterial vaginosis had decreased levels of *Lactobacillus* and other bacteria such as *Gardnerella* and *Prevotella* proliferated (Chen H. et al., 2021; Onderdonk et al., 2016). mNGS revealed bacterial *Gardnerella* and fungal Candida co-infections. mNGS technology can accurately detect this difference in microbial composition, providing a basis for diagnosing and understanding the etiology of bacterial vaginosis (Coudray and Madhivanan, 2020). Relationship between vaginal microbiota and HPV infection: the vaginal micro biostructure of HPV-infected women is significantly different from that of uninfected women, with a decrease in *Lactobacilli* and an increase in aerobic and anaerobic bacteria. mNGS analysis showed that these changes in the microbial composition may affect host immune function, thereby increasing the risk of persistent HPV infection (Santella et al., 2022; Lebeau et al., 2022; Zhang Y. et al., 2022). Dynamic changes in the vaginal microbiota during pregnancy: mNGS was used to track and monitor the changes in vaginal microbiota in pregnant women. The results showed that the microbial composition of the predominance of *Lactobacilli* changed significantly with the progress of pregnancy, which was conducive to preventing complications during pregnancy (Saraf et al., 2021; Smith and Ravel, 2017). Effect of probiotic treatment on vaginal microbiome: the effects of taking probiotic supplements on the vaginal microbiota of women were evaluated using mNGS technology. The results showed that probiotic intervention could significantly increase the abundance of *Lactobacilli* and improve the abnormal microbial composition, which provided a basis for applying probiotics in treating vaginitis and other diseases (Chee et al., 2020; Yang et al., 2020) mNGS can affect our understanding of microbial-related diseases, such as bacterial vaginosis or other reproductive tract infections. mNGS classifies BV into different subtypes based on microbiome characteristics (such as bacterial community composition and functional genes), guiding precise treatment (Zhang et al., 2018). In addition, the causes of some reproductive tract infections (such as atypical vaginitis) remain unknown and may be related to uncultured microorganisms or viruses. mNGS can detect that vaginal bacteriophages (such as *Caudovirales*) may regulate the stability of the bacterial community (Gonzalez-Serrano et al., 2020).

It boasts broad-spectrum detection capabilities, enabling the testing of samples for all microorganisms without bias, including viruses, bacteria, fungi, and parasites. In contrast, traditional methods are typically limited to detecting one specific microorganism at a time (Diao et al., 2022), avoiding a prespecified detection range. The technology does not require a preset detection range, which means it can detect unexpected pathogens, thus enhancing the comprehensiveness of diagnostic outcomes (Diao et al., 2022). Furthermore, the mNGS technology can also be used to detect and study the percentage of host DNA contamination in the vaginal microbial community (Table 4). Therefore, mNGS has apparent advantages in diagnosing LRI with unknown causes and co-infection. Additionally, mNGS provides rapid detection, delivering results relatively quickly, which is crucial for rapidly diagnosing severe infections (Gu et al., 2019; Lv et al., 2024).

**Table 4:** Host DNA contamination percentages in vaginal microbiome studies by using mNGS method.

**Number of samples**	**Site**	**Sequence platform**	**Host DNA**	**References**
71	Vaginal	DNBSEQ-T7	65.53% −99.82%	Wang T. et al., 2024
15	Vaginal	Oxford nanopore	>90%	Mike et al., 2022
351	Vaginal	Metagenomic sequencing	95.5%	Wei et al., 2024
2	Vaginal	Oxford nanopore technologies	97.25%	Ong et al., 2022
6	Cervical	Illumina NextSeq500	93%	Arroyo Mühr et al., 2021
23	Cervical and vaginal	Illumina MiSeq	61.3%	Kaelin et al., 2022

The long-term advantage of mNGS lies in its continuous technological iteration and potential for cross disciplinary applications. With the further reduction of sequencing costs and the increase of throughput, mNGS will promote the comprehensive popularization of precision medicine, achieve personalized diagnosis and early screening and diagnosis. The integration of multiple omics and artificial intelligence will deepen the analysis of complex biological systems, assist in the study of disease mechanisms and target discovery. In the field of public health, mNGS's real-time pathogen monitoring capability can significantly improve the efficiency of global infectious disease prevention and control. The platform-based nature of mNGS makes it the cornerstone of future life science research and the development of the biotechnology industry, providing sustained power for human health and sustainable social development.

#### 3.2.2 The challenges of mNGS both technical and clinical

While mNGS technology offers many benefits, it still faces certain limitations. The application of mNGS technology in clinical diagnostics presents several challenges. Firstly, sample collection, transportation, and potential contamination can introduce errors in the diagnostic process, necessitating stringent quality-control measures to ensure sample integrity (George et al., 2021). Secondly, the vast amount of data produced by mNGS requires specialized bioinformatics tools and expertise for analysis, which can pose a challenge for some laboratories (Edward and Handel, 2021). Additionally, the high cost of mNGS compared to some traditional microbial detection methods may limit its widespread adoption in certain areas (George et al., 2021). Furthermore, even when pathogens are identified, determining their pathogenicity remains challenging and requires a comprehensive evaluation incorporating clinical information and other laboratory test results. Lastly, the standardization and validation of mNGS technology is an evolving field, and further studies and clinical data are needed to establish standardized operating procedures and validation methods (George et al., 2021).

mNGS has many advantages in microbial detection and is becoming increasingly widely used, but there are still many challenges in the actual application process. Firstly, the vast and complex amount of data is one of the main challenges faced by mNGS (Chiu and Miller, 2019). Metagenomic sequencing can generate massive amounts of short DNA fragment sequence data (Lei et al., 2022), which includes genetic information of all microorganisms in the sample. However, extracting valuable information from massive amounts of data and providing accurate explanations is a considerable challenge. In addition, the composition and structure of microbial communities are often very complex (Guo et al., 2021), and the interactions between different microorganisms are also complex (Handel et al., 2021), making data analysis and interpretation more difficult. For example, if we conducted mNGS on a blood sample from a patient suspected of having a complex infection. This sample may contain genetic material from various microorganisms, including bacteria, viruses, fungi, and parasites. Secondly, quality control and data standardization are another important challenge (Han et al., 2019; Bal et al., 2018). Due to the limitations of sequencing technology and the complexity of samples, raw data often has quality issues, such as low-quality sequences, sequencing errors, and host contamination (Lin et al., 2023; Ji et al., 2020). These issues will affect the accuracy and reliability of the data, which in turn will affect subsequent analysis and interpretation. In addition, there is currently a lack of unified mNGS data analysis and interpretation standards, and different research teams may use various methods and parameters, resulting in inconsistent and difficult-to-compare results.

Otherwise, the content of host DNA in vaginal samples is often very high, which leads to many host DNA sequences in sequencing results (Chen H. et al., 2024). These host DNA sequences occupy a large amount of sequencing resources and make the proportion of microbial DNA sequences relatively low, thereby increasing the difficulty of data analysis and interpretation (Mo et al., 2022). To overcome this challenge, researchers often need to perform host sequence filtering on sequencing data to remove interference from host DNA sequences. However, this step carries specific technical difficulties and error risks, excessive filtering may mistakenly delete microbial sequences (especially pathogens with high homology to the host), while insufficient filtering may result in residual host interference, which greatly affects the accuracy of subsequent analysis (Pan et al., 2023).

Moreover, the presence of host DNA may also lead to masking or contamination of microbial DNA sequences. For example, when using molecular biology techniques such as PCR to detect pathogens causing vaginal infections, the presence of a large amount of host DNA may mask or interfere with the signals of the pathogens, leading to false-negative or false-positive results. Due to the similarity in sequence between host DNA and microbial DNA, it is sometimes difficult to completely distinguish. This may lead to certain microbial species being misidentified as host sources or specific host DNA sequences incorrectly identified as microbial sources (Kalantar et al., 2020). This masking or contamination phenomenon can seriously affect the accuracy and reliability of mNGS data, causing difficulties for subsequent interpretation and application (Han et al., 2022; Wang et al., 2022).

There are still some issues that we need to pay attention to. Firstly, the cost of next-generation sequencing of metagenomes is relatively high (Mitchell and Simner, 2019). This technology involves a large amount of sequencing work, requiring the use of high-throughput sequencing platforms. The costs of sequencing reagents, equipment, and consumables are relatively high. Moreover, the amount of data generated by mNGS is huge, which requires professional bioinformatics analysts and powerful computing resources for data analysis. The cost in this aspect is also relatively high (Akaçin et al., 2022). These make the cost of mNGS technology much higher than traditional microbial identification methods. The high cost limits the popularization and application of this technology in areas with limited resources or underdeveloped economies.

The accessibility of next-generation sequencing of metagenomes is limited. Despite the continuous development of sequencing technology, metagenomic sequencing still requires professional laboratory equipment, technical personnel, and bioinformatics analysis capabilities. This makes it difficult for researchers and medical institutions to independently conduct metagenomic sequencing experiments, relying on professional institutions or collaborative laboratories (Han et al., 2019; Sun et al., 2022). In addition, data analysis and interpretation also require profound knowledge and experience in bioinformatics (Gökdemir et al., 2022), which is a challenge for nonprofessionals. Many medical institutions lack relevant equipment or talent and need to outsource to third-party laboratories. Extended the detection cycle.

There are limitations in the interpretation of data for next-generation sequencing of metagenomes. Metagenomic sequencing generates a massive amount of short DNA fragment sequence data, which requires a series of complex steps such as preprocessing, assembly, alignment, and annotation to obtain valuable biological information (Wan et al., 2024). However, each of these steps may introduce errors or uncertainties, which can affect the accuracy and reliability of the result. And after obtaining the data, there are still misjudgments of false positives and false negatives (Diao et al., 2023b). False positives may be due to database annotation errors, such as contamination sequences being mistakenly identified as pathogens, false negatives may be due to low abundance pathogens not being sequenced or pathogen genomes not matching the database. It is worth noting that mNGS may detect multiple microorganisms, but it is difficult to distinguish between contaminating bacteria and real pathogenic bacteria (Liu et al., 2022a). For example, oral commensal bacteria in respiratory samples may have a false clinical value.

## 4 Future perspectives

### 4.1 Advancements in bioinformatics tools and computational resources have resulted in the advancement of mNGS technology

The proliferation of “omics” data from large consortia and individual laboratories, in conjunction with bioinformatics tools (Malkaram et al., 2012), underscores the pressing need for enhanced computational resources to facilitate expeditious, large-scale data processing. Concurrently, substantial technological advancements are poised to overcome the critical limitations of current NGS platforms. Despite the shortcomings of NGS, including its inability to resolve complex genomic regions (e.g., highly repetitive or modified sequences), its lack of single-molecule resolution, and its reproducibility challenges stemming from complex protocols, amplification errors, and reagent dependency (Zhang Y. et al., 2024), third-generation sequencing, particularly nanopore technology, offers transformative potential. The device's capacity for ultra-long reads has been demonstrated to facilitate the process of mapping, while its workflow has been streamlined to enhance reproducibility and reduce experimental artifacts. Most critically, nanopore sequencing achieves true single-molecule sequencing, enabling the direct detection of modifications on individual molecules—a breakthrough which is essential for applications such as rigorously assessing the quality of therapeutic mRNAs where modification patterns are paramount. These advancements, in conjunction with the development of advanced computing and sequencing hardware, signify a paradigm shift toward more efficient, accurate, and insightful biological analysis.

### 4.2 Potential clinical applications of mNGS in vaginal health

We analyzed studies published between 2015–2023 that applied mNGS to vaginal microbiome research. Included studies focused on reproductive-age women (18–45 years) with BV or vulvovaginal candidiasis (VVC), excluding immunocompromised individuals. Samples were collected via standardized swabs from the posterior fornix, with DNA extracted for bacterial (16S rRNA), fungal (ITS), and viral (whole-genome) sequencing. Meta-analysis prioritized bacterial taxa (e.g., Lactobacillus, Gardnerella), while fungal/viral data were summarized descriptively due to heterogeneity in reporting.

In the field of vaginal health, mNGS holds potential clinical application value (Chiu and Miller, 2019). The application of sequencing technology is shifting from research to clinical laboratories owing to rapid technological developments and substantially reduced costs. An agnostic, unbiased, and comprehensive method for detection, and taxonomic characterization of microorganisms, has become an attractive strategy (Li et al., 2021). Since 2008, numerous studies from over 20 countries have revealed the practicality of mNGS in the work-up of undiagnosed diseases. mNGS performs well in identifying rare, novel, difficult-to-detect, and coinfected pathogens directly from clinical samples and presents great potential in resistance prediction by sequencing the antibiotic resistance genes, providing new diagnostic evidence that can be used to guide treatment options and improve antibiotic stewardship (Han et al., 2019). Therefore, using mNGS for the diagnosis of vaginitis is an inevitable trend. Firstly, mNGS can be used in the diagnosis and monitoring of various gynecological diseases such as vaginitis, cervicitis and pelvic inflammatory disease. Vaginitis is a common gynecological disease; its pathogens include bacteria, fungi, viruses, and others (Shroff, 2023; 2020). Traditional methods for detecting pathogenic microorganisms often target only a specific pathogen (Liu et al., 2022a), whereas mNGS can detect multiple pathogens simultaneously (Piantadosi et al., 2021), significantly improving the accuracy and efficiency of diagnosis. For instance, a study conducted NGS tests on 89 vaginal swab samples from South Korean women and found that compared with traditional methods, it had better consistency in predicting vaginitis and could detect more types of microorganisms, which is helpful for accurately diagnosing the disease and assessing its severity (Savicheva et al., 2023). Through mNGS testing, doctors can gain a more accurate understanding of the microbial composition and types of pathogens in the patient's vagina, enabling them to develop more precise treatment plans.

Secondly, mNGS can also be used to assess the vaginal microecological balance (Shen et al., 2022). The vaginal microecological balance is one of the essential factors for maintaining female reproductive health (Shen et al., 2022), and its imbalance may lead to various gynecological diseases (Zhang H. et al., 2022). By sequencing and analyzing the microbiota in vaginal samples, mNGS can understand the composition and diversity of the vaginal microecology (Tsang et al., 2024), For instance, studies have shown that through mNGS testing, the potential risks of reduced diversity of vaginal microbiota and increased specific harmful bacteria can be detected in advance, and then corresponding intervention measures can be taken (Savicheva et al., 2023), thus assessing the balance state of the vaginal microecology. This is significant for preventing and treating gynecological diseases (Chang et al., 2023).

In addition, mNGS can also be used for drug resistance analysis of vaginal infections (Chiu and Miller, 2019). With the widespread use of antibiotics, the issue of drug resistance is becoming increasingly severe (Zhang H. et al., 2022). By sequencing and analyzing the drug-resistance genes of pathogenic microorganisms, mNGS can understand their resistance and sensitivity to antibiotics, providing crucial reference information for clinical treatment (Hu et al., 2023).

### 4.3 The broader applications of mNGS

Macrobarcoding, known as metagenomic science, has been extensively applied since it was used for the health sciences realm in 2008 (Gu et al., 2019; Palacios et al., 2008). Metagenomic next-generation sequencing (mNGS) has emerged as a game-changer in clinical diagnostics, revolutionizing pathogen detection beyond conventional culture-based methods. This cutting-edge technology's true strength lies in its ability to comprehensively analyze diverse clinical specimens—from bodily fluids (Gu et al., 2021) and lung tissue (Qian et al., 2020; Li et al., 2018), to cerebrospinal fluid (Wang X. et al., 2024) and even prosthetic joints (Wang et al., 2020) without any prior bias. By simultaneously detecting all microbial signatures (viral, bacterial, fungal, and parasitic), mNGS overcomes the inherent limitations of traditional techniques that require targeted testing approaches. Particularly valuable for identifying rare, novel, or fastidious pathogens, as well as complex co-infections, this method represents a quantum leap in diagnostic microbiology (Diao et al., 2022). In addition to detecting different infections, beyond the application of mNGS for identifying diverse infections, scholars participating in the Human Microbiome Project have dedicated their efforts to mapping microbial communities that play a significant role in human health, particularly in areas such as the gut, oral cavity, skin, and vaginal region (Turnbaugh et al., 2007; Gevers et al., 2012). mNGS techniques are gaining traction in forensic medicine, particularly for resolving various forensic issues; these applications include geolocation and surface analysis (Habtom et al., 2019), identification (Schmedes et al., 2018), biological sex determination (Zhou and Bian, 2018), trace evidence (Robinson et al., 2020) determination of the mode of death and cause of death (Zhang et al., 2019), and postmortem microbiota determination is becoming increasingly common (Metcalf et al., 2013; Pechal et al., 2014).

## 5 Conclusion

The evolution of detection methods has improved the diagnostic accuracy and efficiency of microbial testing, which has had a profound impact on the study of vaginal microbiology, enabling us to more accurately understand the types and composition of vaginal microbes and helping to study the relationship between vaginal microbes and women's health. mNGS has a significant impact on advancing our understanding of the vaginal microbiome and its impact on women's health.

In terms of studying the vaginal microbiome, there are other emerging technologies and methods in development beyond mNGS techniques that may complement or even surpass mNGS. For example, research presents an approach based on a manifold detection framework for studying the dynamics of the vaginal microbiome. Drawing inspiration from single-cell analysis, this methodology seeks to uncover low-dimensional trajectories within a high-dimensional compositional space, enabling the assignment of a score (pseudo-time) to each sample based on its closeness to the BV state (bacterial vaginosis). This approach can reveal the transition path between health status and BV status and accurately quantify the health status of the sample (Tsamir-Rimon and Borenstein, 2023). In addition, an article pointed out that one focus of vaginal microbiome research is to better characterize the vaginal microbiome community state subtypes and apply advanced “omics” techniques to improve the understanding of the pathogenesis of BV (bacterial vaginopathy). These techniques include 16S ribosomal RNA gene sequencing, which has helped identify previously unrecognized members of the vaginal microbiome, such as *Lactobacillus iner, Atopobium vaginae, Sneathia, Leptotrichia, Megasphaera, Dialister*, and *Eggerthela* (Martin and Marrazzo, 2016).

These studies have shown that the composition of the vaginal microbiome is associated with a variety of women's health conditions, including BV and adverse pregnancy outcomes. These studies suggest that other emerging technologies and approaches besides mNGS are emerging as powerful tools to study the vaginal microbiome, and they may provide deeper understanding and more effective diagnostic and therapeutic strategies in the future.

At the same time, some emerging technologies have also solved some problems faced by some mNGS in the detection of vaginal microorganisms. For example, numerous experiments demonstrate that the parallel algorithm of third-generation sequencing technologies has significantly improved runtime, speedup, throughput, and memory usage. When utilized on the most extensive human dataset, the algorithm achieves an impressive speedup of 10.78 × , resulting in a substantial enhancement of throughput on a vast scale, making the third-generation sequencing technologies enable faster and more efficient processing of large-scale genomic datasets. It effectively makes up for the shortcomings of mNGS in processing complex and massive data (Wang and Zhang, 2024).

While genomics studies can identify many potential biomarkers (Gupta and Alfirevic, 2022), these approaches cannot accurately predict the actual biomass of the protein in question, nor can they infer the metabolic activity or function of the cell in the body, as the presence of a gene does not necessarily indicate when or why the protein is being translated. Meta-proteomic and metabolomics are mass spectrometry-based methods that measure proteins and metabolites in clinical samples, including amniotic fluid, cervicovaginal fluid (CVF), urine, serum, and plasma, enabling the collection of detailed microbial and host functional messages (Masson et al., 2023). Additionally, MS-based proteomics provides quantitative and relative protein abundances and the ability to provide actual microbial function, host immune response, and microbe-microbe and microbe-host interactions. This makes it possible to study further all molecular pathways, cellular components, or biological processes that may be over or under-expressed in different conditions (Masson et al., 2023).

**Figure 5:**
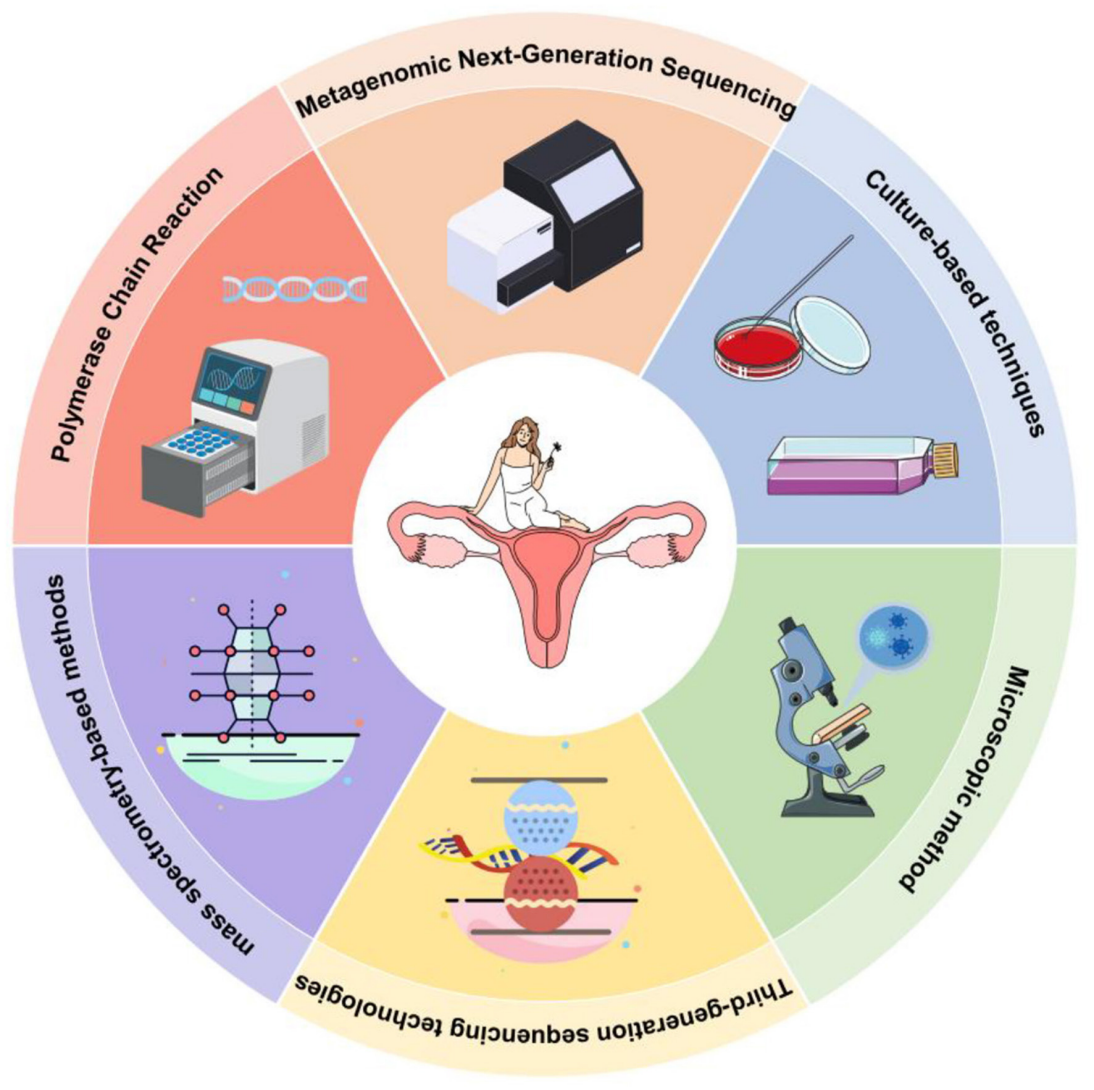
Six standard methods for detecting vaginal microbes. In this review, we analyzed the advantages and disadvantages of mNGS by comparing it with traditional and novel mNGS detection methods.

Although mNGS still faces some challenges and still has some defects in comparison with current technologies, it remains an effective way of identifying and describing the female vaginal microbiome through mNGS based on its mature detection method, skill to detect unexplained disease, the low price compared with other emerging techniques, and combinations of 16S rRNA gene sequencing, qPCR and bacterial culture or shotgun metagenomics together with the analysis of host responses are likely to yield higher quality data to answer some questions (Figure 5). And most of the studies show that mNGS applied in the detection of vaginal microorganisms is more mature, compared to the traditional culture method and microscopy method, as well as the emerging third generation sequencing technology, mNGS still with its broad-spectrum detection ability, and the advantages of the fast speed in the female genital tract microbial detection field has an irreplaceable position. With the continuous improvement of bioinformatics tools and computational resources, mNGS-related techniques are also advancing, enabling mNGS to provide a more comprehensive and unbiased view of microbial communities, becoming a valuable approach to study vaginal health and disease, it is expected to increase our in vaginal microbiome and its impact on women's health understand play an increasingly important role.

## References

[B1] (2020) Vaginitis in nonpregnant patients: ACOG practice bulletin, number 215. Obstet. Gynecol. 135, e1–e17. 10.1097/AOG.0000000000003604. 31856123

[B2] Abbasi I. A. Hess L. W. Johnson T. R. McFadden E. Chernow B. (1985). Leukocyte esterase activity in the rapid detection of urinary tract and lower genital tract infections in obstetric patients. Am. J. Perinatol. 2, 311–313. 10.1055/s-2007-999977 4052183

[B3] Abou Chacra L. Benatmane A. Iwaza R. Ly C. Alibar S. Armstrong N.. (2023). Culturomics reveals a hidden world of vaginal microbiota with the isolation of 206 bacteria from a single vaginal sample. Arch. Microbiol. 206:20. 10.1007/s00203-023-03742-2 38095693 PMC10721685

[B4] Adamczak A. M. Werblińska A. Jamka M. Walkowiak J. (2024). Maternal-foetal/infant interactions-gut microbiota and immune health. Biomedicines 12:490. 10.3390/biomedicines12030490 38540103 PMC10967760

[B5] Akaçin İ. Ersoy Ş. Doluca O. Güngörmüşler M. (2022). Comparing the significance of the utilization of next generation and third generation sequencing technologies in microbial metagenomics. Microbiol. Res. 264:127154. 10.1016/j.micres.2022.127154 35961096

[B6] Amarasinghe S. L. Su S. Dong X. Zappia L. Ritchie M. E. Gouil Q.. (2020). Opportunities and challenges in long-read sequencing data analysis. Genome Biol. 21:30. 10.1186/s13059-020-1935-5 32033565 PMC7006217

[B7] Arango-Sabogal J. C. Dubuc J. Krug C. Denis-Robichaud J. Dufour S. (2019). Accuracy of leukocyte esterase test, endometrial cytology and vaginal discharge score for diagnosing postpartum reproductive tract health status in dairy cows at the moment of sampling, using a latent class model fit within a Bayesian framework. Prev. Vet. Med. 162, 1–10. 10.1016/j.prevetmed.2018.11.003 30621886

[B8] Aronson J. K. Ferner R. E. (2017). Biomarkers - a general review. Curr. Protoc. Pharmacol. 76:9.23.1–9.23.17. 10.1002/cpph.19 28306150

[B9] Arroyo Mühr L. S. Dillner J. Ure A. E. Sundström K. Hultin E. (2021). Comparison of DNA and RNA sequencing of total nucleic acids from human cervix for metagenomics. Sci. Rep. 11:18852. 10.1038/s41598-021-98452-4 34552145 PMC8458301

[B10] Artika I. M. Dewi Y. P. Nainggolan I. M. Siregar J. E. Antonjaya U. (2022). Real-time polymerase chain reaction: current techniques, applications, and role in COVID-19 diagnosis. Genes 13:2387. 10.3390/genes13122387 36553654 PMC9778061

[B11] Arya M. Shergill I. S. Williamson M. Gommersall L. Arya N. Patel H. R.. (2005). Basic principles of real-time quantitative PCR. Expert Rev. Mol. Diag. 5, 209–219. 10.1586/14737159.5.2.209 15833050

[B12] Athanasopoulou K. Boti M. A. Adamopoulos P. G. Skourou P. C. Scorilas A. (2021). Third-generation sequencing: the spearhead towards the radical transformation of modern genomics. Life 12:30. 10.3390/life12010030 35054423 PMC8780579

[B13] Avershina E. Frisli T. Rudi K. (2013). *De novo* semi-alignment of 16S rRNA gene sequences for deep phylogenetic characterization of next generation sequencing data. Microb. Environ. 28, 211–216. 10.1264/jsme2.ME12157 23603801 PMC4070667

[B14] Baker G. C. Smith J. J. Cowan D. A. (2003). Review and re-analysis of domain-specific 16S primers. J. Microbiol. Methods 55, 541–555. 10.1016/j.mimet.2003.08.009 14607398

[B15] Bal A. Pichon M. Picard C. Casalegno J. S. Valette M. Schuffenecker I.. (2018). Quality control implementation for universal characterization of DNA and RNA viruses in clinical respiratory samples using single metagenomic next-generation sequencing workflow. BMC Infect. Dis. 18:537. 10.1186/s12879-018-3446-5 30373528 PMC6206636

[B16] Barousse M. M. Van Der Pol B. J. Fortenberry D. Orr D. Fidel P. L. Jr. (2004). Vaginal yeast colonisation, prevalence of vaginitis, and associated local immunity in adolescents. Sex Transm. Infect. 80, 48–53. 10.1136/sti.2002.003855 14755036 PMC1758371

[B17] Blanco-Míguez A. Beghini F. Cumbo F. McIver L. J. Thompson K. N. Zolfo M.. (2023). Extending and improving metagenomic taxonomic profiling with uncharacterized species using MetaPhlAn 4. Nat. Biotechnol. 41, 1633–1644. 10.1038/s41587-023-01688-w 36823356 PMC10635831

[B18] Bradford L. L. Ravel J. (2016). The vaginal mycobiome: a contemporary perspective on fungi in women's health and diseases. Virulence 8, 342–351. 10.1080/21505594.2016.1237332 27657355 PMC5411243

[B19] Brinkmann A. Ulm S. L. Uddin S. Frster S. Seifert D. Oehme R.. (2021). AmpliCoV: rapid whole-genome sequencing using multiplex PCR amplification and real-time oxford nanopore MinION sequencing enables rapid variant identification of SARS-CoV-2. Front. Microbiol. 12:651151. 10.3389/fmicb.2021.651151 34276587 PMC8281033

[B20] Brooks J. P. Buck G. A. Chen G. Diao L. Edwards D. J. Fettweis J. M.. (2017a). Changes in vaginal community state types reflect major shifts in the *microbiome*. Microb. Ecol. Health Dis. 28:1303265. 10.1080/16512235.2017.1303265 28572753 PMC5443090

[B21] Brooks J. P. Edwards D. J. Blithe D. L. Fettweis J. M. Serrano M. G. Sheth N. U.. (2017b). Effects of combined oral contraceptives, depot medroxyprogesterone acetate and the levonorgestrel-releasing intrauterine system on the vaginal microbiome. Contraception 95, 405–413. 10.1016/j.contraception.2016.11.006 27913230 PMC5376524

[B22] Ceccarani C. Foschi C. Parolin C. D'Antuono A. Reports M. J. S. (2019). Diversity of vaginal microbiome and metabolome during genital *infections*. Sci. Rep. 9:14095. 10.1038/s41598-019-50410-x 31575935 PMC6773718

[B23] Chacko M. R. Kozinetz C. A. Hill R. Collins K. Dunne M. Hergenroeder A. C.. (1996). Leukocyte esterase dipstick as a rapid screening test for vaginitis and cervicitis. J. Pediat. Adolesc. Gynecol. 9, 185–189. 10.1016/S1083-3188(96)70028-1 8957772

[B24] Chang L. Qiu L. Lei N. Zhou J. Guo R. Gao F.. (2023). Characterization of fecal microbiota in cervical cancer patients associated with tumor stage and prognosis. Front. Cell. Infect. Microbiol. 13:1145950. 10.3389/fcimb.2023.1145950 36909733 PMC9995373

[B25] Chee W. J. Y. Chew S. Y. Than L. T. L. (2020). Vaginal microbiota and the potential of *Lactobacillus* derivatives in maintaining vaginal health. Microb. Cell. Fact. 19:203. 10.1186/s12934-020-01464-4 33160356 PMC7648308

[B26] Chen H. Zhang Y. Zheng J. Shi L. He Y. Niu Y.. (2021). Application of mNGS in the etiological diagnosis of thoracic and abdominal infection in patients with end-stage liver *disease*. Front. Cell. Infect. Microbiol. 11:741220. 10.3389/fcimb.2021.741220 35071029 PMC8766839

[B27] Chen H. Zheng Y. Zhang X. Liu S. Yin Y. Guo Y.. (2024). Clinical evaluation of cell-free and cellular metagenomic next-generation sequencing of infected body fluids. J. Adv. Res. 55, 119–129. 10.1016/j.jare.2023.02.018 36889461 PMC10770109

[B28] Chen M. Cai Y. Wang L. Jiang Y. Qian J. Qin J.. (2024). Metagenomic next-generation sequencing testing from the perspective of clinical benefits. Clin. Chim. Acta 553:117730. 10.1016/j.cca.2023.117730 38141936

[B29] Chen X. Lu Y. Chen T. Li R. (2021). The female vaginal microbiome in health and bacterial vaginosis. Front. Cell. Infect. Microbiol. 11:631972. 10.3389/fcimb.2021.631972 33898328 PMC8058480

[B30] Cheng V. C. Yew W. W. Yuen K. Y. (2005). Molecular diagnostics in tuberculosis. Eur. J. Clin. Microbiol. Infect. Dis. 24, 711–720. 10.1007/s10096-005-0039-1 16283213

[B31] Chiu C. Y. Miller S. A. (2019). Clinical metagenomics. Nat. Rev. Genet. 20, 341–355. 10.1038/s41576-019-0113-7 30918369 PMC6858796

[B32] Clarridge J. E. (2004). Impact of 16S rRNA gene sequence analysis for identification of bacteria on clinical microbiology and infectious diseases. Clin. Microbiol. Rev. 17, 840–62. 10.1128/CMR.17.4.840-862.2004 15489351 PMC523561

[B33] Coleman J. S. Gaydos C. A. (2018). Molecular diagnosis of bacterial vaginosis: an Update. J. Clin. Microbiol. 56:e00342–18. 10.1128/JCM.00342-18 29769280 PMC6113459

[B34] Collins S. L. Mcmillan A. Seney S. Veer C. V. D. Kort R. Sumarah M. W.. (2017). *Promising prebiotic candidate established by evaluation of lactitol, lactulose*, raffinose, and oligofructose for maintenance of a *Lactobacillus*-dominated vaginal microbiota. Appl. Environ. Microbiol. 84, e02200–e02217. 10.1128/AEM.02200-17 29269494 PMC5812932

[B35] Coudray M. S. Madhivanan P. (2020). Bacterial vaginosis-a brief synopsis of the literature. Eur. J. Obstet. Gynecol. Reprod. Biol. 245, 143–148. 10.1016/j.ejogrb.2019.12.035 31901667 PMC6989391

[B36] Cummings L. A. Kurosawa K. Hoogestraat D. R. SenGupta D. J. Candra F. Doyle M.. (2016). Clinical next generation sequencing outperforms standard microbiological culture for characterizing polymicrobial samples. Clin. Chem. 62, 1465–1473. 10.1373/clinchem.2016.258806 27624135

[B37] Danby C. S. Althouse A. D. Hillier S. L. Wiesenfeld H. C. (2021). Nucleic acid amplification testing compared with cultures, gram stain, and microscopy in the diagnosis of vaginitis. J. Lower Genit. Tract Dis. 25, 76–80. 10.1097/LGT.0000000000000576 33347046

[B38] Demkin V. V. Koshechkin S. I. Slesarev A. A. (2017). Novel real-time PCR assay for highly specific detection and quantification of vaginal lactobacilli. Mol. Cell. Probes. 32, 33–39. 10.1016/j.mcp.2016.11.006 27890610

[B39] Diao Z. Han D. Zhang R. Li J. (2021). Metagenomics next-generation sequencing tests take the stage in the diagnosis of lower respiratory tract infections. J. Adv. Res. 38, 201–212. 10.1016/j.jare.2021.09.012 35572406 PMC9091713

[B40] Diao Z. Han D. Zhang R. Li J. (2022). Metagenomics next-generation sequencing tests take the stage in the diagnosis of lower respiratory tract infections. J. Adv. Res. 38, 201–212. 35572406 10.1016/j.jare.2021.09.012 PMC9091713

[B41] Diao Z. Lai H. Han D. Yang B. Zhang R. Validation L.. (2023a). of a Metagenomic next-generation sequencing assay for lower respiratory pathogen detection. Microbiol. Spectr. 11:e0381222. 10.1128/spectrum.03812-22 36507666 PMC9927246

[B42] Diao Z. Zhang Y. Chen Y. Han Y. Chang L. Ma Y.. (2023b). Assessing the quality of metagenomic next-generation sequencing for pathogen detection in lower respiratory infections. Clin. Chem. 69, 1038–1049. 10.1093/clinchem/hvad072 37303219

[B43] Drake T. E. Maibach H. I. (1973). Candida and candidiasis. 1. *Cultural conditions, epidemiology and pathogenesis*. Postgrad. Med. 53, 83–87. 10.1080/00325481.1973.11713368 4569505

[B44] Drell T. Lillsaar T. Tummeleht L. Simm J. Aaspõllu A. Väin E.. (2013). *Characterization of the vaginal micro- and mycobiome in asymptomatic reproductive-age Estonian* women. PLoS ONE 8:e54379. 10.1371/journal.pone.0054379 23372716 PMC3553157

[B45] Dubnau D. Smith I. Morell P. Marmur J. (1965). Gene conservation in *Bacillus* species. I. *Conserved genetic and nucleic acid base sequence homologies*. Proc. Natl. Acad. Sci. U.S.A. 54, 491–498. 10.1073/pnas.54.2.491 4956287 PMC219694

[B46] Edward P. Handel A. S. (2021). Metagenomic next-generation sequencing for infectious disease diagnosis: a review of the literature with a focus on pediatrics. J. Pediatr. Infect. Dis. Soc. 10, S71–S7. 10.1093/jpids/piab104 34951466

[B47] Erokhin V. E. Minyuk G. S. Gordienko A. P. Kapranov S. V. (2022). Dynamics of luminescence characteristics of Haematococcus lacustris cultures in different cultivation conditions. Luminescence. 37, 455–462. 10.1002/bio.4194 35029025

[B48] Forney L. J. Gajer P. Williams C. J. Schneider G. M. Koenig S. S. McCulle S. L.. (2010). Comparison of self-collected and physician-collected vaginal swabs for microbiome analysis. J. Clin. Microbiol. 48, 1741–1748. 10.1128/JCM.01710-09 20200290 PMC2863907

[B49] Fredricks D. N. Fiedler T. L. Thomas K. K. Mitchell C. M. Marrazzo J. M. (2009). Changes in vaginal bacterial concentrations with intravaginal metronidazole therapy for bacterial vaginosis as assessed by quantitative *PCR*. J. Clin. Microbiol. 55:307. 10.1128/JCM.01384-08 19144794 PMC2650913

[B50] Gauthier N. P. G. Chorlton S. D. Krajden M. Manges A. R. (2023). Agnostic sequencing for detection of viral pathogens. Clin. Microbiol. Rev. 36:e0011922. 10.1128/cmr.00119-22 36847515 PMC10035330

[B51] George S. Pal A. C. Gagnon J. Timalsina S. Singh P. Vydyam P.. (2021). Evidence for SARS-CoV-2 spike protein in the urine of COVID-19 patients. Kidney360 2, 924–936. 10.34067/KID.0002172021 35373072 PMC8791366

[B52] Gevers D. Knight R. Petrosino J. F. Huang K. McGuire A. L. Birren B. W.. (2012). The human microbiome project: a community resource for the healthy human microbiome. PLoS Biol. 10:e1001377. 10.1371/journal.pbio.1001377 22904687 PMC3419203

[B53] Gökdemir F. Işeri Ö. D. Sharma A. Achar P. N. Eyidogan F. (2022). Metagenomics next generation sequencing (mNGS): an exciting tool for early and accurate diagnostic of fungal pathogens in plants. J. Fungi. 8:1195. 10.3390/jof8111195 36422016 PMC9699264

[B54] Goldacre M. J. Milne L. J. Watt B. Loudon N. Vessey M. P. (2010). *Prevalence of Yeast and fungi other than Candida albicans in the vagina of normal young women*. Br. J. Obstet. Gynaecol. 88, 596–600. 10.1111/j.1471-0528.1981.tb01214.x 7248216

[B55] Gonzalez-Serrano R. Dunne M. Rosselli R. Martin-Cuadrado A.-B.. (2020). *Alteromonas* myovirus V22 represents a new genus of marine bacteriophages requiring a tail fiber chaperone for host recognition. mSystems 5:e00217–20. 10.1128/msystems.00217-20 32518192 PMC7289586

[B56] Goodwin S. Mcpherson J. D. McCombie W. R. (2016). Coming of age: ten years of next-generation sequencing technologies. Nat. Rev. Genet. 17, 333–351. 10.1038/nrg.2016.49 27184599 PMC10373632

[B57] Gu W. Deng X. Lee M. Sucu Y. D. Arevalo S. Stryke D.. (2021). Rapid pathogen detection by metagenomic next-generation sequencing of infected body fluids. Nat. Med. 27, 115–124. 10.1038/s41591-020-1105-z 33169017 PMC9020267

[B58] Gu W. Miller S. Chiu C. Y. (2019). Clinical metagenomic next-generation sequencing for pathogen detection. Annu Rev Pathol. 14, 319–338. 10.1146/annurev-pathmechdis-012418-012751 30355154 PMC6345613

[B59] Guan W. Dong S. Wang Z. Jiao J. Wang X. (2023). Impact of a Lactobacillus dominant cervical microbiome, based on 16S-FAST profiling, on the reproductive outcomes of IVF patients. Front. Cell. Infect. Microbiol. 13:1059339. 10.3389/fcimb.2023.1059339 37305412 PMC10250658

[B60] Guo Y. Li H. Chen H. Li Z. Ding W. Wang J.. (2021). Metagenomic next-generation sequencing to identify pathogens and cancer in lung biopsy tissue. EBioMedicine 73:103639. 10.1016/j.ebiom.2021.103639 34700283 PMC8554462

[B61] Gupta J. K. Alfirevic A. (2022). Systematic review of preterm birth multi-omic biomarker studies. 24, 1–24. 10.1017/erm.2022.13 35379367 PMC9884789

[B62] Gwinn M. MacCannell D. Armstrong G. L. (2019). Next-generation sequencing of infectious pathogens. Jama 321, 893–894. 10.1001/jama.2018.21669 30763433 PMC6682455

[B63] Gwinn M. MacCannell D. R. Khabbaz R. F. (2017). Integrating advanced molecular technologies into public health. J. Clin. Microbiol. 55, 703–714. 10.1128/JCM.01967-16 28031438 PMC5328438

[B64] Habtom H. Pasternak Z. Matan O. Azulay C. Gafny R. Jurkevitch E.. (2019). Applying microbial biogeography in soil forensics. Forensic Sci. Int. Genet. 38, 195–203. 10.1016/j.fsigen.2018.11.010 30447564

[B65] Han D. Diao Z. Lai H. Han Y. Xie J. Zhang R.. (2022). Multilaboratory assessment of metagenomic next-generation sequencing for unbiased microbe detection. J. Adv. Res. 38, 213–222. 10.1016/j.jare.2021.09.011 35572414 PMC9091723

[B66] Han D. Li R. Shi J. Tan P. Zhang R. Liquid L.. (2020). Biopsy for infectious diseases: a focus on microbial cell-free DNA sequencing. Theranostics 10, 5501–5513. 10.7150/thno.45554 32373224 PMC7196304

[B67] Han D. Li Z. Li R. Tan P. Zhang R. Li J.. (2019). mNGS in clinical microbiology laboratories: on the road to maturity. Crit. Rev. Microbiol. 45, 668–685. 10.1080/1040841X.2019.1681933 31691607

[B68] Handel A. S. Muller W. J. Planet P. J. (2021). Metagenomic next-generation sequencing (mNGS): SARS-CoV-2 as an example of the technology's potential pediatric infectious disease applications. J Pediatric Infect Dis Soc. 10, S69–S70. 10.1093/jpids/piab108 34951468 PMC8755271

[B69] Harshitha R. Arunraj D. R. (2021). Real-time quantitative PCR: a tool for absolute and relative quantification. Biochem. Mol. Biol. Educ. 49, 800–812. 10.1002/bmb.21552 34132460

[B70] Heikema A. P. Horst-Kreft D. Boers S. A. Jansen R. Hiltemann S. D. de Koning W.. (2020). Comparison of Illumina versus Nanopore 16S rRNA Gene Sequencing of the human nasal microbiota. Genes 11:1105. 10.3390/genes11091105 32967250 PMC7565314

[B71] Heinze T. Riedewald S. Saling E. (1989). Determination of vaginal pH by pH indicator strip and by pH micro electrode. J. Perinat. Med. 17, 477–479. 10.1515/jpme.1989.17.6.477 2635728

[B72] Hok T. T. Loen L. K. (1967). *Comparative bacteriology of the endocervical mucus*. Short Commun. 98, 781–783. 10.1016/0002-9378(67)90193-7 6027706

[B73] Hong K. H. Hong S. K. Cho S. I. Ra E. Han K. H. Kang S. B.. (2016). Analysis of the vaginal microbiome by next-generation sequencing and evaluation of its performance as a clinical diagnostic tool in vaginitis. Ann. Lab. Med. 36, 441–449. 10.3343/alm.2016.36.5.441 27374709 PMC4940487

[B74] Hu X. Zhao Y. Han P. Liu S. Liu W. Mai C.. (2023). Novel clinical mNGS-Based machine learning model for rapid antimicrobial susceptibility testing of acinetobacter baumannii. J. Clin. Microbiol. 61:e0180522. 10.1128/jcm.01805-22 37022167 PMC10204632

[B75] Huang Y. Zheng W. Gan W. Zhang T. (2023). Chlamydia psittaci pneumonia: a clinical analysis of 12 patients. Ann. Trans. Med. 11:144. 10.21037/atm-22-6624 36846017 PMC9951019

[B76] Hurley R. De Louvois J. (1979). Candida *vaginitis*. Postgrad. Med. J. 55, 645–647. 10.1136/pgmj.55.647.645 523355 PMC2425644

[B77] Ignyś I. Szachta P. Gałecka M. Schmidt M. Pazgrat-Patan M. (2014). Methods of analysis of gut microorganism–actual state of knowledge. Ann. Agric. Environ. Med. 21, 799–803. 10.5604/12321966.1129936 25528923

[B78] Indelli P. F. Ghirardelli S. Violante B. Amanatullah D. F. (2021). Next generation sequencing for pathogen detection in periprosthetic joint infections. EFORT Open Rev. 6, 236–244. 10.1302/2058-5241.6.200099 34040801 PMC8142595

[B79] Janda J. M. Abbott S. L. (2007). 16S rRNA gene sequencing for bacterial identification in the diagnostic laboratory: pluses, perils, and pitfalls. J. Clin. Microbiol. 45, 2761–2764. 10.1128/JCM.01228-07 17626177 PMC2045242

[B80] Jespers V. Menten J. Smet H. Poradosú S. Abdellati S. D. Verhelst R.. (2012). Quantification of bacterial species of the vaginal microbiome in different groups of women, using nucleic acid amplification *tests*. BMC Microbiol. 12:83. 10.1186/1471-2180-12-83 22647069 PMC3418157

[B81] Ji X. C. Zhou L. F. Li C. Y. Shi Y. J. Wu M. L. Zhang Y.. (2020). Reduction of Human DNA contamination in clinical cerebrospinal fluid specimens improves the sensitivity of metagenomic next-generation sequencing. J. Mol. Neurosci. 70, 659–666. 10.1007/s12031-019-01472-z 32002752

[B82] Jung H. Winefield C. Bombarely A. Prentis P. Waterhouse P. (2019). Tools and strategies for long-read sequencing and *De Novo* assembly of plant *genomes. Trends Plant Sci*. 24, 700–724. 10.1016/j.tplants.2019.05.003 31208890

[B83] Kaelin E. A. Skidmore P. T. Łaniewski P. Holland L. A. Chase D. M. Herbst-Kralovetz M. M.. (2022). Cervicovaginal DNA virome alterations are associated with genital inflammation and microbiota composition. mSystems 7:e0006422. 10.1128/msystems.00064-22 35343798 PMC9040584

[B84] Kalantar K. L. Carvalho T. de Bourcy C. F. A. Dimitrov B. Dingle G. Egger R.. (2020). IDseq-An open source cloud-based pipeline and analysis service for metagenomic pathogen detection and monitoring. GigaScience 9:giaa111. 10.1093/gigascience/giaa111 33057676 PMC7566497

[B85] Kalia N. Singh J. Kaur M. (2020). Microbiota in vaginal health and pathogenesis of recurrent vulvovaginal infections: a critical review. Ann. Clin. Microbiol. Antimicrob. 19:5. 10.1186/s12941-020-0347-4 31992328 PMC6986042

[B86] Kalra A. Palcu C. T. Sobel J. D. Akins R. A. (2007). Bacterial vaginosis: culture- and PCR-based characterizations of a complex polymicrobial disease's pathobiology. Curr. Infect. Dis. Rep. 9, 485–500. 10.1007/s11908-007-0074-4 17999885

[B87] Kero K. Hieta N. Kallonen T. Ahtikoski A. Laine H. K. Rautava J.. (2023). Optimal sampling and analysis methods for clinical diagnostics of vaginal microbiome. Eur. J. Clin. Microbiol. Infect. Dis. 42, 201–208. 10.1007/s10096-022-04545-x 36624297 PMC9837015

[B88] Khaki P. Rahimi Zarchi F. Moradi Bidhendi S. Gharakhani M. (2023). Application of a multiplex PCR assay for molecular identification of pathogenic and non-pathogenic leptospires based on lipL32 and 16S rRNA genes. Arch. Razi Inst. 78, 413–418. 10.22092/ARI.2022.359211.2388 37312705 PMC10258299

[B89] Klein D. (2002). Quantification using real-time PCR technology: applications and limitations. Trends Mol. Med. 8, 257–260. 10.1016/S1471-4914(02)02355-9 12067606

[B90] Kubista M. Andrade J. M. Bengtsson M. Forootan A. Jonák J. Lind K.. (2006). The real-time polymerase chain reaction. Mol. Asp. Med. 27, 95–125. 10.1016/j.mam.2005.12.007 16460794

[B91] Lazarevic V. Gaïa N. Girard M. Mauffrey F. Ruppé E. Schrenzel J.. (2022). Effect of bacterial DNA enrichment on detection and quantification of bacteria in an infected tissue model by metagenomic next-generation sequencing. ISME Commun. 2:122. 10.1038/s43705-022-00208-2 37938717 PMC9792467

[B92] Lebeau A. Bruyere D. Roncarati P. Peixoto P. Hervouet E. Cobraiville G.. (2022). HPV infection alters vaginal microbiome through down-regulating host mucosal innate peptides used by Lactobacilli as amino acid sources. Nat. Commun. 13:1076. 10.1038/s41467-022-28724-8 35228537 PMC8885657

[B93] Lecuit M. Eloit M. (2014). The diagnosis of infectious diseases by whole genome next generation sequencing: a new era is opening. Front. Cell. Infect. Microbiol. 4:25. 10.3389/fcimb.2014.00025 24639952 PMC3944390

[B94] Lee C. J. Shin W. Song M. Shin S. S. Park Y. Srikulnath K.. (2023). Comparison of digital PCR platforms using the molecular marker. Genom. Inform. 21:e24. 10.5808/gi.23008 37704210 PMC10326530

[B95] Lefterova M. I. Suarez C. J. Banaei N. Pinsky B. A. (2015). Next-generation sequencing for infectious disease diagnosis and management: a report of the association for molecular pathology. J. Mol. Diagn. 17, 623–634. 10.1016/j.jmoldx.2015.07.004 26433313

[B96] Lei W. Fei-Zhou Z. Jing C. Shu-Xian L. Xi-Ling W. Lan-Fang T.. (2022). Pseudomembranous necrotizing laryngotracheobronchitis due to *Mycoplasma* pneumoniae: a case report and literature review. BMC Infect. Dis. 22:183. 10.1186/s12879-022-07160-5 35197010 PMC8867838

[B97] Li C. Zhao R. Yang H. Ren L. (2023). Construction of bone hypoxic microenvironment based on bone-on-a-chip platforms. Int. J. Mol. Sci. 24:6999. 10.3390/ijms24086999 37108162 PMC10139217

[B98] Li H. Gao H. Meng H. Wang Q. Li S. Chen H.. (2018). Detection of pulmonary infectious pathogens from lung biopsy tissues by metagenomic next-generation sequencing. Front. Cell. Infect. Microbiol. 8:205. 10.3389/fcimb.2018.00205 29988504 PMC6026637

[B99] Li N. Cai Q. Miao Q. Song Z. Fang Y. Hu B.. (2021). High-throughput metagenomics for identification of pathogens in the clinical settings. Small Methods 5:2000792. 10.1002/smtd.202000792 33614906 PMC7883231

[B100] Lian Q. Song X. Yang J. Wang L. Xu P. Wang X.. (2024). Alterations of lung microbiota in lung transplant recipients with pneumocystis jirovecii pneumonia. Respir. Res. 25:125. 10.1186/s12931-024-02755-9 38486264 PMC10941442

[B101] Lin W. Xie F. Li X. Yang R. Lu J. Ruan Z.. (2023). Diagnostic performance of metagenomic next-generation sequencing and conventional microbial culture for spinal infection: a retrospective comparative study. Eur. Spine J. 32, 4238–4245. 10.1007/s00586-023-07928-6 37689612

[B102] Liu H. Zhang Y. Yang J. Liu Y. Chen J. (2022a). Application of mNGS in the etiological analysis of lower respiratory tract infections and the prediction of drug resistance. Microbiol. Spectr. 10:e0250221. 10.1128/spectrum.02502-21 35171007 PMC8849087

[B103] Liu Y. Ghaffari M. H. Ma T. Tu Y. (2024). Impact of database choice and confidence score on the performance of taxonomic classification using Kraken2. aBIOTECH 5, 465–475. 10.1007/s42994-024-00178-0 39650139 PMC11624175

[B104] Liu Y. Zhang R. Yao B. Yang J. Ge H. Zheng S.. (2022b). Metagenomics next-generation sequencing provides insights into the causative pathogens from critically ill patients with pneumonia and improves treatment strategies. Front. Cell. Infect. Microbiol. 12:1094518. 10.3389/fcimb.2022.1094518 36710980 PMC9880068

[B105] Lopez M. J. (2013). Gendered vulnerabilities: women, HIV, and preventive interventions in sub-Saharan Africa. J. Int. Womens Stud. 14, 103–117.

[B106] Lv T. Zhao Q. Liu J. Wang S. Wu W. Miao L.. (2024). Utilizing metagenomic next-generation sequencing for pathogen detection and diagnosis in lower respiratory tract infections in real-world clinical practice. Infection 52, 625–636. 10.1007/s15010-024-02185-1 38368306

[B107] Mårdh P. A. Novikova N. Niklasson O. Bekassy Z. Skude L. (2003). Leukocyte esterase activity in vaginal fluid of pregnant and non-pregnant women with vaginitis/vaginosis and in controls. Infect. Dis. Obstet. Gynecol. 11, 19–26. 10.1155/S1064744903000036 12839629 PMC1852264

[B108] Ma B. Forney L. J. Ravel J. (2012). Vaginal microbiome: rethinking health and disease. Ann. Rev. Microbiol. 66, 371–389. 10.1146/annurev-micro-092611-150157 22746335 PMC3780402

[B109] Mahajan G. Doherty E. To T. Sutherland A. Grant J. Junaid A.. (2022). Vaginal microbiome-host interactions modeled in a human vagina-on-a-chip. Microbiome 10:201. 10.1186/s40168-022-01400-1 36434666 PMC9701078

[B110] Mahler L. Niehs S. P. Martin K. Weber T. Scherlach K. Hertweck C.. (2021). Highly parallelized droplet cultivation and prioritization of antibiotic producers from natural microbial communities. eLife. 10:e64774. 10.7554/eLife.64774.sa2 33764297 PMC8081529

[B111] Maksimovic Celicanin M. Haahr T. Humaidan P. Skafte-Holm A. (2024). Vaginal dysbiosis - the association with reproductive outcomes in IVF patients: a systematic review and meta-analysis. Curr Opin Obstet Gynecol. 36, 155–164. 10.1097/GCO.0000000000000953 38597377 PMC11062609

[B112] Malkaram S. A. Hassan Y. I. Zempleni J. (2012). Online tools for bioinformatics analyses in nutrition sciences. Adv. Nutr. 3, 654–665. 10.3945/an.112.002477 22983844 PMC3648747

[B113] Marcellino R. B. Morsella C. G. Cano D. Paolicchi F. A. (2015). [Efficiency of bacteriological culture and the immunofluorescent assay to detect Campylobacter fetus in bovine genital fluids]. Rev. Argent. Microbiol. 47, 183–189. 10.1016/j.ram.2015.03.008 26187267

[B114] Margolles A. Ruiz L. (2021). Methods for isolation and recovery of bifidobacteria. Methods Mol. Biol. 2278, 1–12. 10.1007/978-1-0716-1274-3_1 33649943

[B115] Margulies M. Egholm M. Altman W. E. Attiya S. Bader J. S. Bemben L. A.. (2006). Corrigendum: gnome sequencing in microfabricated high-density picolitre reactors. Nature 441:120. 10.1038/nature03959 16056220 PMC1464427

[B116] Martin D. H. Marrazzo J. M. (2016). The vaginal microbiome: current understanding and future directions. J. Infect. Dis. 214, S36–S41. 10.1093/infdis/jiw184 27449871 PMC4957511

[B117] Masson L. Wilson J. Amir Hamzah A. S. Tachedjian G. Payne M. (2023). Advances in mass spectrometry technologies to characterize cervicovaginal microbiome functions that impact spontaneous preterm birth. Am. J. Reprod. Immunol. 90:e13750. 10.1111/aji.13750 37491925

[B118] Mattei V. Murugesan S. Al Hashmi M. Mathew R. James N. Singh P.. (2019). Evaluation of methods for the extraction of microbial DNA from vaginal swabs used for microbiome studies. Front. Cell. Infect. Microbiol. 9:197. 10.3389/fcimb.2019.00197 31245304 PMC6563847

[B119] Metcalf J. L. Wegener Parfrey L. Gonzalez A. Lauber C. L. Knights D. Ackermann G.. (2013). A microbial clock provides an accurate estimate of the postmortem interval in a mouse model system. eLife 2:e01104. 10.7554/eLife.01104 24137541 PMC3796315

[B120] Michael T. P. Jupe F. Bemm F. Motley S. T. Sandoval J. P. Lanz C.. (2018). High contiguity Arabidopsis thaliana genome assembly with a single nanopore flow *cell*. Nat. Commun. 9:541. 10.1038/s41467-018-03016-2 29416032 PMC5803254

[B121] Mike M. Janine Z. Jana P. Adrian V. Ekkehard S. Oliwia M.. (2022). Evaluation of microbiome enrichment and host DNA depletion in human vaginal samples using Oxford Nanopore's adaptive sequencing. Sci. Rep. 12:4000. 10.1038/s41598-022-08003-8 35256725 PMC8901746

[B122] Mitchell S. L. Simner P. J. (2019). Next-generation sequencing in clinical microbiology: are we there yet? Clin. Lab. Med. 39, 405–418. 10.1016/j.cll.2019.05.003 31383265

[B123] Mo B. Sendker J. Herrmann F. Nowak S. Hensel A. (2022). Aqueous extract from Equisetum arvense stimulates the secretion of Tamm-Horsfall protein in human urine after oral intake. Phytomedicine 104:154302. 10.1016/j.phymed.2022.154302 35809378

[B124] Moreno I. Simon C. (2019). Deciphering the effect of reproductive tract microbiota on human reproduction. Reprod. Med. Biol. 18, 40–50. 10.1002/rmb2.12249 30655720 PMC6332752

[B125] Mortaki D. Gkegkes I. D. Psomiadou V. Blontzos N. Prodromidou A. Lefkopoulos F.. (2020). Vaginal microbiota and human papillomavirus: a systematic review. J. Turk. Ger. Gynecol. Assoc. 21, 193–200. 10.4274/jtgga.galenos.2019.2019.0051 31564082 PMC7495129

[B126] Muzny C. A. Cerca N. Elnaggar J. H. Taylor C. M. Sobel J. D. Van Der Pol B. (2023). State of the art for diagnosis of bacterial vaginosis. J. Clin. Microbiol. 61:e0083722. 10.1128/jcm.00837-22 37199636 PMC10446871

[B127] Nell R. J. Zoutman W. H. Versluis M. van der Velden P. A. (2022). Generic Multiplex digital PCR for accurate quantification of T Cells in copy number stable and unstable DNA samples. Methods Mol. Biol. 2453, 191–208. 10.1007/978-1-0716-2115-8_12 35622328 PMC9761916

[B128] Nori W. Vaginal B. H. H. (2023). Microbes confounders and implications on women's health. World J. Clin. Cases 11, 2119–2122. 10.12998/wjcc.v11.i9.2119 36998952 PMC10044948

[B129] Noval Rivas M. Burton O. T. Wise P. Zhang Y. Q. Hobson S. A. Garcia Lloret M.. (2013). A microbiota signature associated with experimental food allergy promotes allergic sensitization and anaphylaxis. J. Allergy Clin. Immunol. 131, 201–212. 10.1016/j.jaci.2012.10.026 23201093 PMC3860814

[B130] Nygaard A. B. Tunsj H. S. Meisal R. Charnock C. J. S. R. (2020). A preliminary study on the potential of Nanopore MinION and Illumina MiSeq 16S rRNA gene sequencing to characterize building-dust microbiomes. Sci. Rep. 10:3209. 10.1038/s41598-020-59771-0 32081924 PMC7035348

[B131] Oerlemans E. Ahannach S. Wittouck S. Dehay E. Boeck I. D. Ballet N.. (2022). Impacts of menstruation, community type, and an oral yeast probiotic on the vaginal microbiome. mSphere 7, e00239–e00222. 10.1128/msphere.00239-22 36102507 PMC9599324

[B132] Oh J. Byrd A. L. Deming C. Conlan S. Kong H. H. Segre J. A.. (2014). Biogeography and individuality shape function in the human skin metagenome. Nature 514, 59–64. 10.1038/nature13786 25279917 PMC4185404

[B133] O'Hanlon D. E. Come R. A. Moench T. R. (2019). Vaginal pH measured in vivo: lactobacilli determine pH and lactic acid concentration. BMC Microbiol. 19:13. 10.1186/s12866-019-1388-8 30642259 PMC6332693

[B134] O'Hanlon D. E. Moench T. R. Cone R. A. (2013). *Vaginal pH and Microbicidal Lactic acid when Lactobacilli dominate the* microbiota. PLoS ONE 8:e80074. 10.1371/journal.pone.0080074 24223212 PMC3819307

[B135] Onderdonk A. B. Delaney M. L. Fichorova R. N. (2016). The human microbiome during bacterial vaginosis. Clin. Microbiol. Rev. 29, 223–238. 10.1128/CMR.00075-15 26864580 PMC4786887

[B136] Ong C. T. Ross E. M. Boe-Hansen G. B. Turni C. Hayes B. J. Tabor A. E.. (2022). Technical note: overcoming host contamination in bovine vaginal metagenomic samples with nanopore adaptive sequencing. *J*. Anim. Sci. 100:skab344. 10.1093/jas/skab344 34791313 PMC8722758

[B137] Paavonen J. Brunham R. C. (2019). Bacterial vaginosis and desquamative inflammatory vaginitis. 380:1089. 10.1056/NEJMc1900134 30865816

[B138] Palacios G. Druce J. Du L. Tran T. Birch C. Briese T.. (2008). A new arenavirus in a cluster of fatal transplant-associated diseases. N. Engl. J. Med. 358, 991–998. 10.1056/NEJMoa073785 18256387

[B139] Pan Y. Shang G. Li J. Zhang Y. Liu J. Ji Y.. (2023). Case report: a novel IRF2BP2 mutation in an IEI patient with recurrent infections and autoimmune disorders. Front. Immunol. 14:967345. 10.3389/fimmu.2023.967345 37350971 PMC10282741

[B140] Pashaian M. M. Oganesian G. G. (2011). [Isolation and characterization of vaginal lactobacilli producing hydrogen peroxide]. Zh. Mikrobiol. Epidemiol. Immunobiol. 2011, 90–93. 22308738

[B141] Patil M. J. Nagamoti J. M. Metgud S. C. (2012). Diagnosis of trichomonas vaginalis from vaginal specimens by wet mount microscopy, in pouch TV culture system, and PCR. J. Glob. Infect. Dis. 4, 22–25. 10.4103/0974-777X.93756 22529623 PMC3326953

[B142] Pechal J. L. Crippen T. L. Benbow M. E. Tarone A. M. Dowd S. Tomberlin J. K.. (2014). The potential use of bacterial community succession in forensics as described by high throughput metagenomic sequencing. Int. J. Legal Med. 128, 193–205. 10.1007/s00414-013-0872-1 23749255

[B143] Peebles K. Velloza J. Balkus J. E. McClelland R. S. Barnabas R. V. (2019). High global burden and costs of bacterial vaginosis: a systematic review and meta-analysis. Sex. Transm. Dis. 46, 304–311. 10.1097/OLQ.0000000000000972 30624309

[B144] Peng X. Y. Wu J. T. Shao C. L. Li Z. Y. Chen M. Wang C. Y.. (2021). Co-culture: stimulate the metabolic potential and explore the molecular diversity of natural products from microorganisms. Mar. Life Sci. Technol. 3, 363–374. 10.1007/s42995-020-00077-5 37073292 PMC10077301

[B145] Piantadosi A. Mukerji S. S. Ye S. Leone M. J. Freimark L. M. Park D.. (2021). Enhanced virus detection and metagenomic sequencing in patients with meningitis and encephalitis. mBio 12:e0114321. 10.1128/mBio.01143-21 34465023 PMC8406231

[B146] Preksha G. Yesheswini R. Srikanth C. V. (2021). Cell culture techniques in gastrointestinal research: methods, possibilities and challenges. Indian J. Pathol. Microbiol. 64, S52–S57. 10.4103/IJPM.IJPM_933_20 34135138

[B147] Punzón-Jiménez P. Labarta E. (2021). The impact of the female genital tract microbiome in women health and reproduction: a review. J. Assist. Reprod. Genet. 38, 2519–2541. 10.1007/s10815-021-02247-5 34110573 PMC8581090

[B148] Qian Y. Y. Wang H. Y. Zhou Y. Zhang H. C. Zhu Y. M. Zhou X.. (2020). Improving pulmonary infection diagnosis with metagenomic next generation sequencing. Front. Cell. Infect. Microbiol. 10:567615. 10.3389/fcimb.2020.567615 33585263 PMC7874146

[B149] Ravel J. Gajer P. Abdo Z. Schneider G. M. Koenig S. S. K. Mcculle S. L.. (2011). *Vaginal microbiome of reproductive-age* women. Proc. Natl. Acad. Sci. U.S.A. 108, 4680–4687. 10.1073/pnas.1002611107 20534435 PMC3063603

[B150] Roberts R. J. Carneiro M. O. Schatz M. C. (2013). *The advantages of SMRT sequencing*. Genome Biol. 14:405. 10.1186/gb-2013-14-7-405 23822731 PMC3953343

[B151] Robinson J. M. Pasternak Z. Mason C. E. Elhaik E. (2020). Forensic applications of microbiomics: a review. Front. Microbiol. 11:608101. 10.3389/fmicb.2020.608101 33519756 PMC7838326

[B152] Roy P. Mirza T. T. Paul S. K. Shamsi S. Khan M. K. Begum M. F.. (2023). Comparison of wet mount microscopy and giemsa staining to PCR in the diagnosis of vaginal trichomoniasis in a Tertiary Level Hospital of Bangladesh. Mymensingh Med. J. 32, 348–354. 37002744

[B153] Sahu B. Singh S. D. Behera B. K. Panda S. K. Das A. Parida P. K.. (2019). Rapid detection of Salmonella contamination in seafoods using multiplex PCR. Braz. J. Microbiol. 50, 807–816. 10.1007/s42770-019-00072-8 31006836 PMC6863201

[B154] Santella B. Schettino M. T. Franci G. De Franciscis P. Colacurci N. Schiattarella A.. (2022). Microbiota and HPV: The role of viral infection on vaginal microbiota. J. Med. Virol. 94, 4478–4484. 10.1002/jmv.27837 35527233 PMC9544303

[B155] Santiago G. L. Cools P. Verstraelen H. Trog M. Missine G. El Aila N.. (2011). Longitudinal study of the dynamics of vaginal microflora during two consecutive menstrual cycles. PLoS ONE 6:e28180. 10.1371/journal.pone.0028180 22140538 PMC3227645

[B156] Saraf V. S. Sheikh S. A. Ahmad A. Gillevet P. M. Bokhari H. Javed S.. (2021). Vaginal microbiome: normalcy vs dysbiosis. Arch. Microbiol. 203, 3793–3802. 10.1007/s00203-021-02414-3 34120200

[B157] Savicheva A. M. (2023). Molecular testing for the diagnosis of bacterial vaginosis. Int. J. Mol. Sci. 25:449. 10.3390/ijms25010449 38203620 PMC10779368

[B158] Savicheva A. M. Krysanova A. A. Budilovskaya O. V. Spasibova E. V. Khusnutdinova T. A. Shalepo K. V.. (2023). Vaginal microbiota molecular profiling in women with bacterial vaginosis: a novel diagnostic *tool*. *Int. J. Mol. Sci*. 24:15880. 10.3390/ijms242115880 37958862 PMC10649576

[B159] Scarano C. Veneruso I. De Simone R. R. Di Bonito G. Secondino A. D'Argenio V.. (2024). The Third-Generation Sequencing Challenge: Novel Insights for the Omic Sciences. Biomolecules. 14:568. 10.3390/biom14050568 38785975 PMC11117673

[B160] Scher J. U. Sczesnak A. Longman R. S. Segata N. Ubeda C. Bielski C.. (2013). Expansion of intestinal Prevotella copri correlates with enhanced susceptibility to arthritis. eLife 2:e01202. 10.7554/eLife.01202 24192039 PMC3816614

[B161] Schmedes S. E. Woerner A. E. Novroski N. M. M. Wendt F. R. King J. L. Stephens K. M.. (2018). Targeted sequencing of clade-specific markers from skin microbiomes for forensic human identification. Forensic Sci. Int. Genet. 32, 50–61. 10.1016/j.fsigen.2017.10.004 29065388

[B162] Schmidt H. Hansen J. G. (2001). Validity of wet-mount bacterial morphotype identification of vaginal fluid by phase-contrast microscopy for diagnosis of bacterial vaginosis in family practice. APMIS. 109, 589–594. 10.1034/j.1600-0463.2001.d01-179.x 11878711

[B163] Schmidt K. Cybulski Z. Roszak A. Grabiec A. Talaga Z. Urbański B.. (2015). Combination of microbiological culture and multiplex PCR increases the range of vaginal microorganisms identified in cervical cancer patients at high risk for bacterial vaginosis and vaginitis. Ginekol. Polska. 86, 328–334. 10.17772/gp/2417 26117968

[B164] Sharma M. Chopra C. Mehta M. Sharma V. Mallubhotla S. Sistla S.. (2021). An insight into vaginal microbiome techniques. Life 11:1229. 10.3390/life11111229 34833105 PMC8623751

[B165] Shen L. Zhang W. Yuan Y. Zhu W. Shang A. (2022). Vaginal microecological characteristics of women in different physiological and pathological period. Front. Cell. Infect. Microbiol. 12:959793. 10.3389/fcimb.2022.959793 35937699 PMC9354832

[B166] Shroff S. (2023). Infectious vaginitis, cervicitis, and pelvic inflammatory disease. Med. Clin. North Am. 107, 299–315. 10.1016/j.mcna.2022.10.009 36759099

[B167] Smith S. B. Ravel J. (2017). The vaginal microbiota, host defence and reproductive physiology. J. Physiol. 595, 451–463. 10.1113/JP271694 27373840 PMC5233653

[B168] Sofie T. Michiel O. D. B. Bram B. Sascha T. Vincent S. Van H. J. D.. (2017). Comparative evaluation of four bacteria-specific primer Pairs for 16S rRNA gene *surveys*. *Front. Microbiol*. 8: 494. 10.3389/fmicb.2017.00494 28400755 PMC5368227

[B169] Song S. D. Acharya K. D. Zhu J. E. Deveney C. M. Walther-Antonio M. R. S. Tetel M. J.. (2020). Daily vaginal microbiota fluctuations associated with natural hormonal cycle contraceptives diet and exercise. mSphere 5:e00593–20. 10.1128/msphere.00593-20 32641429 PMC7343982

[B170] Sood A. Ray P. Angrup A. (2022). Antimicrobial susceptibility testing of anaerobic bacteria: in routine and research. Anaerobe 75:102559. 10.1016/j.anaerobe.2022.102559 35417767

[B171] Spiegel C. A. (1989). Vaginitis/vaginosis. Clin. Lab. Med. 9, 525–533. 10.1016/S0272-2712(18)30616-4 2676321

[B172] Subramaniam A. Kumar R. Cliver S. P. Zhi D. Szychowski J. M. Abramovici A.. (2016). Vaginal microbiota in pregnancy: evaluation based on vaginal flora, birth outcome, and race. Am. J. Perinatol. 33, 401–408. 10.1055/s-0035-1565919 26479170 PMC5166555

[B173] Sun L. Zhang S. Yang Z. Yang F. Wang Z. Li H.. (2022). Clinical application and influencing factor analysis of metagenomic next-generation sequencing (mNGS) in ICU patients with sepsis. Front. Cell. Infect. Microbiol. 12:905132. 10.3389/fcimb.2022.905132 35909965 PMC9326263

[B174] Tomás M. S. Bru E. Nader-Macías M. E. (2003). Comparison of the growth and hydrogen peroxide production by vaginal probiotic lactobacilli under different culture conditions. Am. J. Obstet. Gynecol. 188, 35–44. 10.1067/mob.2003.123 12548193

[B175] Tsamir-Rimon M. Borenstein E. A. (2023). A manifold-based framework for studying the dynamics of the vaginal microbiome. npj Biofilms Microbiomes. 9:102. 10.1038/s41522-023-00471-8 38102172 PMC10724123

[B176] Tsang H.-F. Cheung Y.-S. Allen Yu C.-A. Sammy Chan C.-S. Thomas Wong C.-B. Aldrin Yim K.-Y.. (2024). Menstrual blood as a diagnostic specimen for human papillomavirus genotyping and genital tract infection using next-generation sequencing as a novel diagnostic tool. Diagnostics 14:686. 10.3390/diagnostics14070686 38611599 PMC11012019

[B177] Turnbaugh P. J. Ley R. E. Hamady M. Fraser-Liggett C. M. Knight R. Gordon J. I.. (2007). The human microbiome project. Nature 449, 804–810. 10.1038/nature06244 17943116 PMC3709439

[B178] van den Tweel M. M. van der Struijs S. van den Munckhof E. H. A. Boers K. E. (2022). The relationship between vaginal pH and bacterial vaginosis as diagnosed using qPCR in an asymptomatic subfertile population. Arch. Gynecol. Obstet. 306, 1787–1793. 10.1007/s00404-022-06764-1 36083500

[B179] van Dijk E. L. Jaszczyszyn Y. Naquin D. Thermes C. (2018). The third revolution in sequencing technology. Trends Genet. 34, 666–681. 10.1016/j.tig.2018.05.008 29941292

[B180] Vartoukian S. R. Palmer R. M. Wade W. G. (2010). Cultivation of a synergistetes strain representing a previously uncultivated lineage. Environ. Microbiol. 12, 916–928. 10.1111/j.1462-2920.2009.02135.x 20074237 PMC2916210

[B181] Villaseca R. Ovalle A. Amaya F. Labra B. Escalona N. Lizana P.. (2015). [Vaginal infections in a Family Health Clinic in the Metropolitan Region, Chile]. Rev. Chil. Infectol. 32, 30–36. 10.4067/S0716-10182015000200005 25860041

[B182] Wallace M. P. Moodie E. E. (2014). Personalizing medicine: a review of adaptive treatment strategies. Pharmacoepidemiol. Drug Safety 23, 580–585. 10.1002/pds.3606 24700536

[B183] Wan S. Zhou A. Chen R. Fang S. Lu J. Lv N.. (2024). Metagenomics next-generation sequencing (mNGS) reveals emerging infection induced by *Klebsiella pneumoniaeniae*. Int. J. Antimicrob. Agents 63:107056. 10.1016/j.ijantimicag.2023.107056 38081548

[B184] Wan X. Yang Q. Wang X. Bai Y. Liu Z. (2023). Isolation and cultivation of human gut microorganisms: a review. Microorganisms 11:1080. 10.3390/microorganisms11041080 37110502 PMC10141110

[B185] Wang B. Liu W. Liu X. Franks A. E. Teng Y. Luo Y. (2017). Comparative analysis of microbial communities during enrichment and isolation of DDT-degrading bacteria by culture-dependent and -independent methods. Sci. Total Environ. 590–591, 297–303. 10.1016/j.scitotenv.2017.03.004 28274604

[B186] Wang C. Huang Z. Li W. Fang X. Zhang W. (2020). Can metagenomic next-generation sequencing identify the pathogens responsible for culture-negative prosthetic joint infection? BMC Infect. Dis. 20:253. 10.1186/s12879-020-04955-2 32228597 PMC7106575

[B187] Wang H. Wang H. Zhang H. Huang Y. Zhang N. Li W.. (2022). Integration of a multichannel surface plasmon resonance sensor chip and refractive index matching film array for protein detection in human urine. Talanta 246:123533. 10.1016/j.talanta.2022.123533 35550509

[B188] Wang J. Han Y. Feng J. (2019). Metagenomic next-generation sequencing for mixed pulmonary infection diagnosis. BMC Pulmon. Med. 19:252. 10.1186/s12890-019-1022-4 31856779 PMC6921575

[B189] Wang R. Zhang Y. (2024). Accelerating spliced alignment of long RNA sequencing reads using parallel maximal exact match retrieval. Comput. Biol. Med. 175:108542. 10.1016/j.compbiomed.2024.108542 38714048

[B190] Wang T. Li P. Bai X. Tian S. Yang M. Leng D.. (2024). Vaginal microbiota are associated with in vitro fertilization during female infertility. iMeta 3:e185. 10.1002/imt2.185 38898981 PMC11183179

[B191] Wang X. Wang K. Xie F. Han Z. Liu Y. Pan L.. (2023). Protocol of a multicenter, single-blind, randomized, parallel controlled trial evaluating the effect of microbiological rapid on-site evaluation (M-ROSE) guiding anti-infection treatment in patients with severe hospital-acquired pneumonia. Trials 24:552. 10.1186/s13063-023-07570-z 37612723 PMC10464107

[B192] Wang X. Xu H. Qin L. Wang H. Jiang Y. Liu H.. (2024). Metagenomic next-generation sequencing of cerebrospinal fluid reveals etiological and microbiological features in patients with various central nervous system infections. FASEB J. (2024) 38:e23812. 10.1096/fj.202400792R 39041354

[B193] Wei X. Tsai M.-S. Liang L. Jiang L. Hung C.-J. Jelliffe-Pawlowski L.. (2024). Vaginal microbiomes show ethnic evolutionary dynamics and positive selection of *Lactobacillus* adhesins driven by a long-term niche-specific process. Cell Rep. 43:114078. 10.1016/j.celrep.2024.114078 38598334

[B194] Willinger B. (2017). Culture-based techniques. Methods Mol. Biol. 1508, 195–207. 10.1007/978-1-4939-6515-1_10 27837505

[B195] Woese C. R. Fox G. E. (1977). Phylogenetic structure of the prokaryotic domain: the primary kingdoms. Proc. Natl. Acad. Sci. U.S.A. 74, 5088–90. 10.1073/pnas.74.11.5088 270744 PMC432104

[B196] Xiao G. Cai Z. Guo Q. Ye T. Tang Y. Guan P.. (2022). Insights into the unique lung microbiota profile of pulmonary tuberculosis patients using metagenomic next-generation sequencing. Microbiol. Spectr. 10:e0190121. 10.1128/spectrum.01901-21 35196800 PMC8865484

[B197] Yang S. Reid G. Challis J. R. G. Gloor G. B. Asztalos E. Money D.. (2020). Effect of oral probiotic *Lactobacillus rhamnosus* GR-1 and *Lactobacillus reuteri* RC-14 on the vaginal microbiota, cytokines and chemokines in pregnant women. Nutrients 12:368. 10.3390/nu12020368 32019222 PMC7071157

[B198] Zhang H. Jin S. Ji A. Zhang C. Shi S. (2022). Correlation between vaginal microecological status and prognosis of CIN patients with high-risk HPV infection. Biomed. Res. Int. 2022:3620232. 10.1155/2022/3620232 35463993 PMC9023142

[B199] Zhang J. Luo J. Weng X. Zhu Y. Goyal G. Perna F.. (2022). A case report of the metagenomics next-generation sequencing for early detection of central nervous system mucormycosis with successful rescue in patient with recurrent chronic lymphocytic leukemia. Ann. Trans. Med. 10:722. 10.21037/atm-22-2533 35845522 PMC9279813

[B200] Zhang P. Xiao Z. Wang S. Zhang M. Wei Y. Hang Q.. (2018). ZRANB1 is an EZH2 deubiquitinase and a potential therapeutic target in breast cancer. Cell. Rep. 23, 823–837. 10.1016/j.celrep.2018.03.078 29669287 PMC5933875

[B201] Zhang Y. Pechal J. L. Schmidt C. J. Jordan H. R. Wang W. W. Benbow M. E.. (2019). Machine learning performance in a microbial molecular autopsy context: a cross-sectional postmortem human population study. PLoS ONE 14:e0213829. 10.1371/journal.pone.0213829 30986212 PMC6464165

[B202] Zhang Y. Xu X. Yu L. Shi X. Min M. Xiong L.. (2022). Vaginal microbiota changes caused by HPV infection in Chinese women. Front. Cell. Infect. Microbiol. 12:814668. 10.3389/fcimb.2022.814668 35800384 PMC9253274

[B203] Zhang Y. Yan H. Wei Z. Hong H. Huang D. Liu G.. (2024). NanoMUD: profiling of pseudouridine and N1-methylpseudouridine using Oxford Nanopore direct RNA sequencing. Int. J. Biol. Macromol. 270:132433. 10.1016/j.ijbiomac.2024.132433 38759861

[B204] Zhao N. Cao J. Xu J. Liu B. Liu B. Chen D.. (2021). Targeting RNA with next- and third-generation sequencing improves pathogen identification in clinical samples. Adv. Sci. 8:e2102593. 10.1002/advs.202102593 34687159 PMC8655164

[B205] Zheng N. Guo R. Yao Y. Jin M. Cheng Y. Ling Z. (2019). Lactobacillus iners Is Associated with vaginal dysbiosis in healthy pregnant women: a preliminary *study*. *Biomed. Res. Int*. 2019:6079734. 10.1155/2019/6079734 31781627 PMC6855029

[B206] Zhou W. Bian Y. (2018). Thanatomicrobiome composition profiling as a tool for forensic investigation. Forensic Sci. Res. 3, 105–110. 10.1080/20961790.2018.1466430 30483658 PMC6197100

[B207] Zhu H. Zhang H. Xu Y. Laššáková S. Korabečná M. NeuŽil P. (2020). PCR past, present and future. BioTechniques 69, 317–325. 10.2144/btn-2020-0057 32815744 PMC7439763

[B208] Zodzika J. Rezeberga D. Jermakova I. Vasina O. Vedmedovska N. Donders G.. (2011). Factors related to elevated vaginal pH in the first trimester of pregnancy. Acta Obstet. Gynecol. Scand. 90, 41–46. 10.1111/j.1600-0412.2010.01011.x 21275914

[B209] Zozaya-Hinchliffe M. Lillis R. Martin D. H. (2010). Quantitative PCR assessments of bacterial species in women with and without bacterial vaginosis. ASM J. 48, 1812–1819. 10.1128/JCM.00851-09 20305015 PMC2863870

